# Fitting Procedure to Reconstruct the Size Distribution and the Concentration of Silver Colloidal Nanoparticles from UV-Vis Spectra

**DOI:** 10.3390/nano12193302

**Published:** 2022-09-22

**Authors:** Julio Car, Nikša Krstulović

**Affiliations:** Institute of Physics, Bijenička Cesta 46, 10000 Zagreb, Croatia

**Keywords:** colloidal silver nanoparticles, fitting function, UV-Vis spectra, log-normal size distribution reconstruction, concentration of nanoparticles

## Abstract

In this work, a complete fitting procedure of UV-Vis spectra of silver nanoparticles in colloidal solutions is reported. The fitting function, based on the Beer–Lambert law, Mie theory, and log-normal probability distribution of nanoparticles’ sizes, is developed and confirmed by 33 different independent measurements. In order to validate the accuracy of the function’s behavior on different spectra, freely accessible measurements were used, proving that the fitting function works independently of the method of their production—laser or chemical synthesis of nanoparticles. The developed fitting function is, to the best of our knowledge, novel and not based on any conventional spectral analysis approaches like the Mie–Gans procedure. Furthermore, since fitted parameters are all physical, it allows determination of the mode diameter of nanoparticles as well as the standard deviation of the log-normal distribution of sizes. It enables the reconstruction of size distribution of nanoparticles in colloidal solution. Step-by-step derivation of the fitting function is provided with a physical explanation of all parameters. The importance of Lorentzian dependence emerging at the core of Beer–Lambert law is physically discussed and linked to harmonic oscillator behavior of localized surface plasmon resonance of silver nanoparticles in a colloidal solution. Size distribution reconstruction from fitted parameters according to a log-normal distribution function is provided and a concentration calculation is presented.

## 1. Introduction

Pulsed laser ablation in liquids (PLAL) is a versatile physical technique that uses laser radiation to evaporate different materials immersed in liquids in order to get nanoparticles in the relevant size range up to 100 nm [1]. Laser ablation as well as thermal evaporation and condensation, atom deposition and condensation fall under the category of gas-phase preparation methods for nanoparticles’ synthesis. On the other hand, there are liquid-phase methods like chemical reduction, hydrolysis, photolysis, sol-gel methods, and mechanical like milling, ultrasound, and spraying [2]. Silver nanoparticles obtained by laser ablation in liquids and chemical techniques differ mainly in the width of size distribution [3] and need for stabilizing agents to keep the nanoparticle solution stable from agglomeration [4]. While the advantage of laser synthesis is the purity of the method, control of final nanoparticles’ sizes is not yet achieved [5] like in chemical reduction methods [6], which, on the other hand, have disadvantages of chemical impurities. The importance of silver nanoparticles grows as they show excellent properties and applications in catalysis [7], optoelectronics [8], energetics [9], sensorics [10], toxicology [11], nanomedicine [12], cancer nanotechnology [13], antibacterial [14,15], etc. In all of the mentioned applications, the size of statistically distributed nanoparticles has a major role in efficiency and capacity of a given field [16]. Beneficence of localized surface plasmons in the visible part of the spectrum and damping frequency above SPR frequency [17] make silver nanoparticles even more useful, for instance in optical imaging and bio-labelling of molecules. Colorimetric variations of colloidal solution of silver nanoparticles are a direct indicator of chemical activity as well as the size of nanoparticles, since SPR wavelength is directly dependent on the size of nanoparticles [18]. Conventional characterization techniques for sizes and concentrations of synthesized nanoparticles obtained either by laser or chemically involve electron microscopy, atomic force microscopy, dynamic light scattering, resistive pulse sensors, ICP-MS, XRD, etc. [19]. Those techniques are both time consuming and expensive, so a cheap and easily accessible alternative is needed. As was shown in [20], UV-Vis spectroscopy in combination with optical microscopy suffices for determination of the size of nanoparticles in a colloidal solution. Since that model was based on absorbance at SPR wavelength and the volume of the crater left after laser ablation, it gave the characteristic diameter of nanoparticles in a colloidal solution. The approach developed in this work is also based on the Beer–Lambert law although expanded due to redshift of scattered wavelengths and is incorporated into fitting function. Since the fitting function considers entire UV-Vis spectra excluding the interband region where the Beer–Lambert law does not hold, it may serve as a verification method for diameters of nanoparticles obtained by a simple analytical model reported in our previous work [20]. Although there are approaches to determine the size distribution of nanoparticles by UV-Vis spectroscopy solely, they include fitting constants that are not physically defined [21]. The fitting function developed in this work has all parameters physically defined and verified by independent TEM measurements with a given size distribution. Motivation and the need for a fitting function for UV-Vis spectra in the nanoscale range primarily serves the purpose of excluding the need for electron microscopy and additional characterization methods or techniques in order to obtain relevant physical properties of silver nanoparticles in colloidal solutions. Data used in the analysis were the following: five UV-Vis spectra of laser-synthesized nanoparticles measured by authors [20], 25 UV-Vis spectra from commercially available data from the company NanoComposix (San Diego, CA, USA), out of which 10 were NanoXact nanoparticles with citrate surface [22], 10 were BioPure purified from all reactants [23], five were Econix with PVP (polyvinylpyrrolidone) surface [24], and three were randomly chosen from independent authors [25,26,27].

## 2. Computational Methods and Results

UV-Vis spectroscopy is a technique based on absorption and scattering of electromagnetic radiation on nanoparticles in a colloidal solution. The ratio of transmitted and incident intensity of light defines absorbance or optical density, which given versus wavelength gives UV-Vis spectrum. The interaction of electromagnetic radiation with nanoscale matter heavily depends on the size of nanoparticles and the wavelength of incident light as well as on the dielectric functions of nanoparticle material and the surrounding medium. Before an electrodynamical model for the description of optical properties of colloidal nanoparticles is used, a statistical distribution of the nanoparticle system must be defined. According to measurements obtained by TEM, AFM, and DLS, independently [20], it is shown that the size distribution of laser synthesized nanoparticles follows a log-normal distribution function, which is defined with mode (most frequent) diameter and standard deviation of distribution. The basis of the fitting function for UV-Vis spectra developed in this work is the Beer–Lambert law, which states that absorbance of light in a colloidal solution of nanoparticles is linearly dependent on extinction cross section, concentration of nanoparticles, and optical path length. Mathematically, it can be written:

(1) $$A=-\log\left(\frac{I_{t}}{I_{0}}\right)=\frac{1}{\ln\left(10\right)}\sigma_{e}c_{A}l$$

Which follows from the Beer–Lambert law given by $10^{-A}=\frac{I_{t}}{I_{0}}=e^{-\sigma_{e}c_{A}l}$. Absorbance defined by Equation (1) considers both absorption and scattering contributions to cross section giving overall extinction cross section as their sum. Modeled parameters used in the fitting function are enumerated and elaborated as follows:

1. $\sigma_{e}$: Mie scattering theory, developed as analytical solutions of Maxwell equations on obstacles with well-defined boundary conditions, gives expressions for absorption $\sigma_{a}$ and scattering $\sigma_{s}$ cross sections, which for spheres in the dipole limit are:

(2a) $$\sigma_{a}=3\pi^{2}\varepsilon_{m}^{3/2}\frac{\varepsilon_{2}}{{\left(\varepsilon_{1}+2\varepsilon_{m}\right)}^{2}+\varepsilon_{2}^{2}}\frac{D^{3}}{\lambda }=K_{1}\frac{D^{3}}{\lambda }$$

(2b) $$\sigma_{s}=\frac{2}{3}\pi^{5}\varepsilon_{m}^{2}\frac{{\left(\varepsilon_{1}-\varepsilon_{m}\right)}^{2}+\varepsilon_{2}^{2}}{{\left(\varepsilon_{1}+2\varepsilon_{m}\right)}^{2}+\varepsilon_{2}^{2}}\frac{D^{6}}{\lambda^{4}}=K_{2}\frac{D^{6}}{\lambda^{4}}$$

As will be seen, Mie theory dipole terms are sufficient to describe UV-Vis spectra larger than 20 nm and smaller than 90 nm by a developed fitting function.

2. The volume concentration $c_{V}$ of nanoparticles depends on volume of all nanoparticles combined, volume of individual nanoparticles, and the probability of a nanoparticle with a given size appearing in colloidal solution. The probability of a given size nanoparticle appearing in a colloidal solution is given by a log-normal probability density function. At this point, there is a difference between volume concentration $c_{V}$ defined in this way and absorbance concentration $c_{A}$ in the Beer–Lambert law. Although the light of different wavelengths illuminates a colloidal solution with the same concentration of nanoparticles, it seems that the effective diameter of nanoparticles that dominates and substitutes individual contribution of all others is not the same for each wavelength regarding absorption and scattering efficiency. The effective diameter, which given the wavelength of light ‘sees’ is a function that is hypothesized to be Lorentzian-like shaped and that, when multiplied with the above defined volume concentration, gives the absorbance concentration. The source of this function will be discussed later as it seems it originates from redshift of scattering wavelengths due to the size of nanoparticles, which leads to electromagnetic depolarization on the surface of nanoparticles. The fitting function, therefore, consists of the following: dipole absorption $\sigma_{a}$ and scattering $\sigma_{s}$ cross sections, concentration $c_{V}$ as a function of the size of individual nanoparticles in the colloidal solution, an additional Lorentzian-like term for transferring the volume concentration $c_{V}$ into absorbance concentration $c_{A}$ and optical path length l. Individual components are as follows:

(3a) $$\sigma_{e}=K_{1}\frac{D_{j}{}^{3}}{\lambda }+K_{2}\frac{D_{j}{}^{6}}{\lambda^{4}}$$

where $K_{1}$ and $K_{2}$ are defined by (2a) and (2b). Volume concentration is given by:

(3b) $$c_{V}\left(D_{j}\right)=\frac{N_{j}\left(D_{j}\right)}{V_{\mathrm{liq}}}=\frac{V_{c}}{V_{j}V_{\mathrm{liq}}}\cdot p\left(D_{j}\right)=\frac{6V_{c}}{V_{\mathrm{liq}}{\pi D}_{j}^{3}}\cdot p\left(D_{j}\right)=\frac{6V_{c}}{V_{\mathrm{liq}}{\pi D}_{j}^{3}}\cdot \frac{f\left(D_{j}\right)\Delta D}{\int_{D_{\min}}^{D_{\max}}f\left(D\right)\mathrm{dD}}$$

Which is according to the definition in [20] given as product of crater volume $V_{c}$ and probability $p\left(D_{j}\right)$ of occurrence of nanoparticles with diameter $D_{j}$ over the volume of nanoparticles with the same diameter $D_{j}$. This gives a number of nanoparticles $N_{j}\left(D_{j}\right)$ with diameter $D_{j}$. According to the form of the log-normal distribution function, it can be written:

(3c) $$\overset{D_{\max}}{\underset{D_{\min}}{\int }}f\left(D\right)\mathrm{dD}=P$$

(3d) $$f\left(D_{j}\right)=\frac{P}{\sqrt{2\pi }s}\cdot \left(\frac{1}{D_{m}e^{\frac{s^{2}}{2}}}\cdot e^{-\frac{{\left(\left(\ln\left(D_{j}\right)-\ln(D_{m}\right)\right)}^{2}}{2s^{2}}}\right)$$

which gives the size-distribution of nanoparticles in a colloidal solution with parameters mode diameter $D_{m}$ and standard deviation s. Log-normal distribution function is fully described by these two parameters: mode diameter $D_{m}$ and standard deviation s. Equation (3d) is used for size distribution reconstruction with a procedure described in Supplementary Materials S4. Absorbance concentration $c_{A}$ is defined as a product of volume concentration $c_{V}$ and redshift function $r\left(D,\lambda \right)$:

(3e) $$c_{A}=c_{V}\cdot r\left(D,\lambda \right)$$

**Figure 1:**
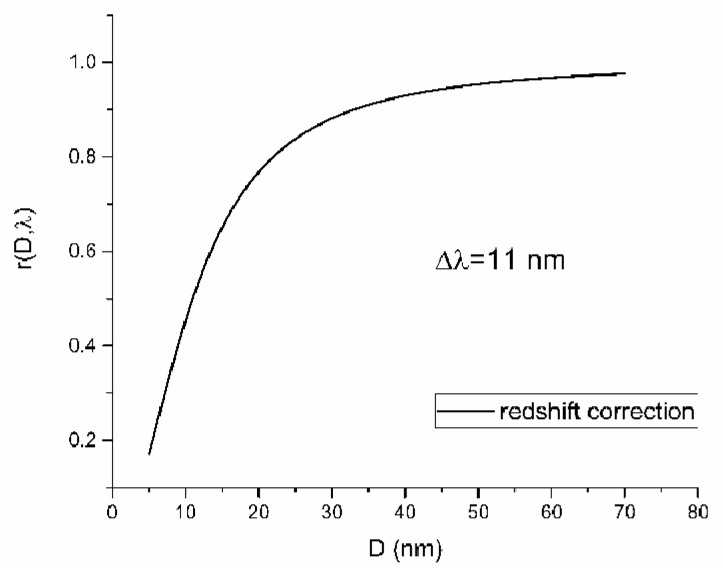
Redshift function vs. diameter of nanoparticles for K=1 and fixed Δλ.

$r\left(D,\lambda \right)$ represents a redshift function, which modifies volume concentration to obtain absorbance concentration. This function was hypothesized to be Lorentzian-like shaped. It has the following form:

(3f) $$r\left(D_{i},\lambda \right)=K\cdot \frac{D_{i}{}^{2}}{D_{i}{}^{2}+{\left(\Delta \lambda \right)}^{2}}$$

where K is a non-dimensional redshift constant in $\left[\frac{\mathrm{nm}}{\mathrm{nm}}\right],$ which can be interpreted as $\left[\frac{\Delta \lambda }{\Delta D}\right]$, $D_{i}$ is the diameter of nanoparticles with a given size, and Δλ is a shift in resonant wavelength from the theoretical one. Theoretical resonance wavelength $\lambda_{0}$ is one for which Frohlich’s condition is satisfied: $\varepsilon_{1}=-2\varepsilon_{m}$ [17]. A graph of the redshift function is shown in Figure 1.

Since the fitting function describes the dependence of absorbance on the wavelength, that is, the UV-Vis spectrum, the independent variable is wavelength. Since both extinction cross section and concentration depend on the diameter of nanoparticles, it is necessary to model diameter by wavelength variable in order to have correct dependence of absorbance on wavelength. It can be done by defining $D_{j}=D\cdot \frac{\lambda }{\lambda_{0}}$ where D is an unknown and undefined diameter value which is not important as a result of a fitting function. Notice that diameter stands as a variable in extinction cross section as well as in concentration and redshift function. It seems that the diameter value that stands as extinction cross section and concentration is not the same as in the redshift function. In the redshift function, the inverse diameter value is modeled and is written as $D_{i}=D\cdot \frac{\lambda_{0}}{\lambda }$. Moreover, the redshift function is modified Lorentzian in form $r\left(D_{i},\lambda \right)=K\cdot \frac{D_{i}{}^{2}}{D_{i}{}^{2}+{\left(\lambda -\lambda_{0}\right)}^{2}}$. Qualitatively, reciprocal dependencies of modeled diameters in the extinction cross section and the log-normal distribution opposite to the redshift function implies the following. Since the modeled diameter in the redshift function regulates the width of the Lorentzian, it seems that for small values of diameter $D_{j}$ in the extinction cross section and log-normal distribution, diameter $D_{i}$ in the redshift function is reciprocally larger, meaning that for nanoparticles of small sizes, the spectral linewidth is reciprocally larger in accordance with [28]. Since all parameters in a fitting function are physical and defined, the same applies for parameter K. The physical explanation of the redshift constant K is that it represents a shift in wavelength by diameter unit and depends on nanoparticle material dielectric properties, size as well as the size distribution of nanoparticles in a colloidal solution. In order to derive a function for K, extinction efficiency given as a ratio of extinction and geometric cross section is examined. For the definition of extinction cross section given by (3a) and the geometric cross section given by $\sigma_{g}=\frac{\pi }{4}D^{2}$, the following is obtained:

(4) $$\left(\frac{\sigma_{e}}{\sigma_{g}}\right)\left(\frac{D}{\lambda }\right)=\frac{4}{\pi }\frac{D}{\lambda }\left(K_{1}+K_{2}{\left(\frac{D}{\lambda }\right)}^{3}\right)$$

If plotted for fixed $K_{1}$, $K_{2},$ and λ, the extinction efficiency as a function of diameter has a minimum. Mathematically, the diameter value for which the minimum is obtained, $D_{\min}$, is given by:

(5) $$\frac{\partial \left(\frac{\sigma_{e}}{\sigma_{g}}\right)}{\partial D}=0\to D_{\min}=-\sqrt[{3}]{\frac{1}{4}}\sqrt[{3}]{\frac{K_{1}}{K_{2}}}\lambda$$

**Figure 2:**
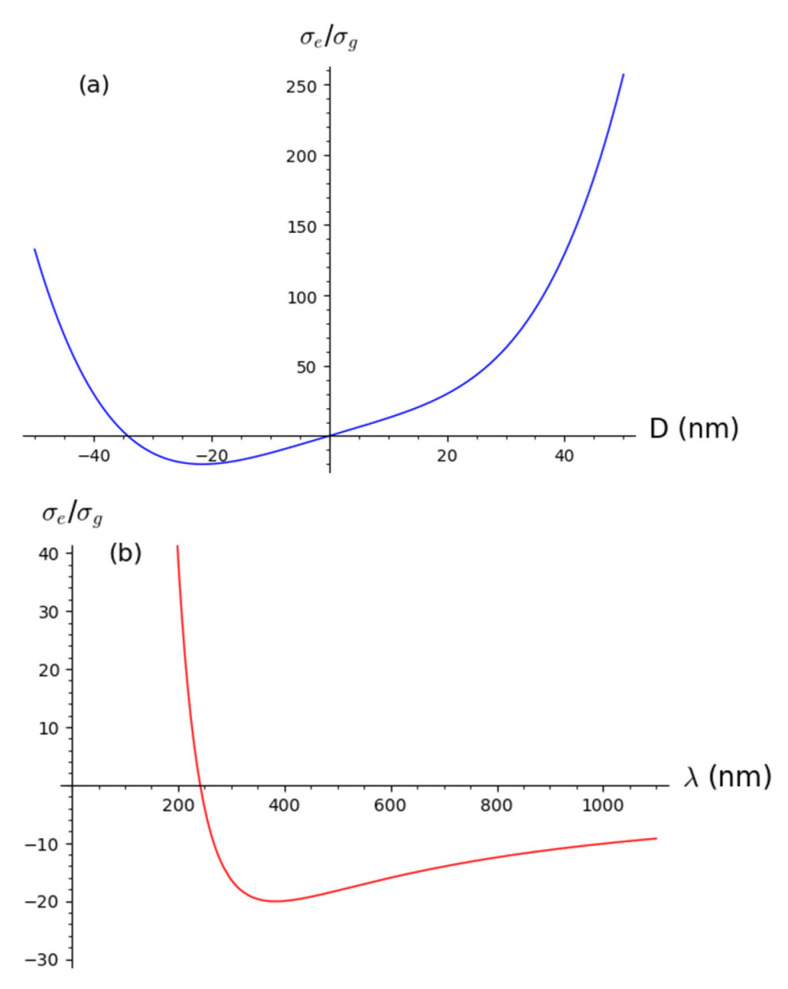
Dependence of extinction to geometric cross section ratio on diameter of nanoparticles (**a**) and on wavelength (**b**).

Figure 2 shows functional dependence $\left(\frac{\sigma_{e}}{\sigma_{g}}\right)\left(\frac{D}{\lambda }\right)\;$ given by Formula (4) on an independent variable D with fixed $K_{1}$, $K_{2}$, and λ corresponding to theoretical SPR (blue curve, (a)) and on an independent variable λ with fixed $K_{1}$, $K_{2}$, and D also corresponding to theoretical SPR (red curve, (b)).

Notice that $D_{\min}<0$ physically does not represent any value characterizing the colloidal solution of nanoparticles. The purpose of this value is, however, meaningful since it represents the mathematical point that is easiest to consider while implementing shift function. Physically, the following must be satisfied:

(6) $$\left(\frac{\sigma_{e}}{\sigma_{g}}\right)\left(D_{0},\lambda_{0}\right)\cdot K\cdot \frac{D^{2}}{{\left(\Delta \lambda \right)}^{2}+D^{2}}=\left(\frac{\sigma_{e}}{\sigma_{g}}\right)\left(D_{\mathrm{SPR}},\lambda_{\mathrm{SPR}}\right)$$

where $\left(\frac{\sigma_{e}}{\sigma_{g}}\right)\left(D_{0},\lambda_{0}\right)$ represents extinction efficiency at theoretical LSPR for which Frohlich condition is satisfied with $D_{0}=-\sqrt[{3}]{\frac{1}{4}}\sqrt[{3}]{\frac{K_{10}}{K_{20}}}\lambda_{0}$, $K\cdot \frac{D^{2}}{{\left(\Delta \lambda \right)}^{2}+D^{2}}$ is redshift function where $\Delta \lambda =\lambda_{\mathrm{SPR}}-\lambda_{0}$, which shifts from theoretical to experimental SPR represented by extinction efficiency $\left(\frac{\sigma_{e}}{\sigma_{g}}\right)\left(D_{\mathrm{SPR}},\lambda_{\mathrm{SPR}}\right)$ with $D_{\mathrm{SPR}}=-\sqrt[{3}]{\frac{1}{4}}\sqrt[{3}]{\frac{K_{1\mathrm{SPR}}}{K_{2\mathrm{SPR}}}}\lambda_{\mathrm{SPR}}$. Now, since K is redshift constant, it should be associated with experimental SPR condition, that is $D_{\mathrm{SPR}}$. In order to determine K, the following equation can be written:

(7) $$\left(\frac{\sigma_{e}}{\sigma_{g}}\right)\left(D_{\mathrm{SPR}},\lambda_{\mathrm{SPR}}\right)\cdot K\cdot \frac{D_{\mathrm{SPR}}{}^{2}}{{\left(\Delta \lambda \right)}^{2}+D_{\mathrm{SPR}}{}^{2}}=\left(\frac{\sigma_{e}}{\sigma_{g}}\right)\left(D_{\mathrm{SPR}},\lambda_{\mathrm{SPR}}\right)$$

Because Equation (6) must hold for each pair $\left(\lambda_{\min},D_{\min}\right)$. This equation then implies that Lorentzian-like term $r\left(D_{\mathrm{SPR}},\lambda \right)=K\cdot \frac{D_{\mathrm{SPR}}{}^{2}}{{\left(\Delta \lambda \right)}^{2}+D_{\mathrm{SPR}}{}^{2}}=1,$ giving:

(8) $$K=1+{\left(\frac{\Delta \lambda }{D_{\mathrm{SPR}}}\right)}^{2}=1+4^{\frac{2}{3}}{\left(\frac{K_{2\mathrm{SPR}}}{K_{1\mathrm{SPR}}}\right)}^{\frac{2}{3}}{\left(\frac{\Delta \lambda }{\lambda_{\mathrm{SPR}}}\right)}^{2}$$

**Figure 3:**
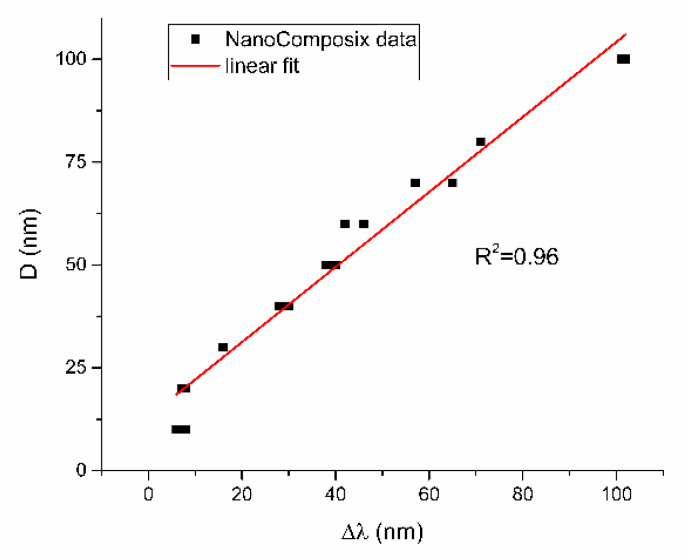
Dependence of mode diameter values obtained by TEM imaging for NanoComposix data on the shift of experimental to theoretical SPR wavelength.

In the obtained formula for the redshift constant K, there is no explicit dependence on the size or size distribution of nanoparticles in colloidal solution, only on dielectric constants, which are incorporated in $K_{1\mathrm{SPR}}$ and $K_{2\mathrm{SPR}}$. Since it is known in theory that redshift depends on the size and size distribution of nanoparticles, these variables must be hidden in the ratio $\frac{\Delta \lambda }{\lambda_{\mathrm{SPR}}}$. Dependence of $\lambda_{\mathrm{SPR}}$ on D is well known to exist [21] and was checked on NanoComposix data (Nanoxact and BioPure nanoparticles). As can be seen in Figure 3, the dependence of reported TEM diameters on the difference between experimental and theoretical SPR wavelength is linear and shown to be: $D\left(\lambda \right)=13+0.91\cdot \left|\lambda_{\mathrm{SPR}}-\lambda_{0}\right|$ for NanoComposix data.

Furthermore, referring to previously published work [20], one can, using equality $c_{V}=c_{A}$ (volume concentration and absorbance concentration are equal), determine K in the following way. Absorbance concentration must be given by:

(9) $$c_{A}\left(D_{j}\right)=c_{A}\cdot p\left(D_{j}\right)=c_{A}\cdot \frac{f\left(D_{j}\right)\Delta D}{\int_{D_{\min}}^{D_{\max}}f\left(D\right)\mathrm{dD}}$$

Since it is based on the number distribution of nanoparticles, so one can imply the following condition:

(10) $$\overset{D_{\max}}{\underset{D_{\min}}{\int }}c_{A}\left(D\right)\mathrm{dD}=\overset{D_{\max}}{\underset{D_{\min}}{\int }}c_{V}\left(D\right)\mathrm{Dd}$$

Condition (10) ensures that the total concentrations obtained by absorbance and volume approach must be equal since the concentration of colloidal nanoparticles is constant. Integrating both sides according to the definition of volume concentration Equation (3b) and absorbance concentration Equation (9), the following is obtained:

(11) $$c_{A}=\frac{6V_{c}}{V_{\mathrm{liq}}\pi }\cdot \frac{1}{D_{m}{}^{3}e^{-\frac{3}{2}s^{2}}}=\frac{6V_{c}}{V_{\mathrm{liq}}\pi }\cdot \frac{1}{{\left(D_{m}e^{-\frac{1}{2}s^{2}}\right)}^{3}}=\frac{6V_{c}}{V_{\mathrm{liq}}\pi }\cdot \frac{1}{D^{3}}=c_{V}$$

where $\langle D\rangle =D_{m}e^{-\frac{1}{2}s^{2}}$ is the volume average diameter and s is the standard deviation of the log-normal distribution. This result states that the substitute diameter for all size distribution of nanoparticles in a colloidal solution is volume average diameter 〈D〉. Notice that Equation (11) is generally based on equality of $c_{A}$ and $c_{V}$ and does not include a redshift function $r\left(D,\lambda \right)$. If, however, a redshift function is included, the following can be written according to Equations (3e) and (10):

(12) $$\overset{D_{\max}}{\underset{D_{\min}}{\int }}c_{A}\left(D\right)\mathrm{dD}=\overset{D_{\max}}{\underset{D_{\min}}{\int }}c_{V}\left(D\right)\cdot K\cdot \frac{D^{2}}{{\left(\Delta \lambda \right)}^{2}+D^{2}}\mathrm{dD}$$

**Figure 4:**
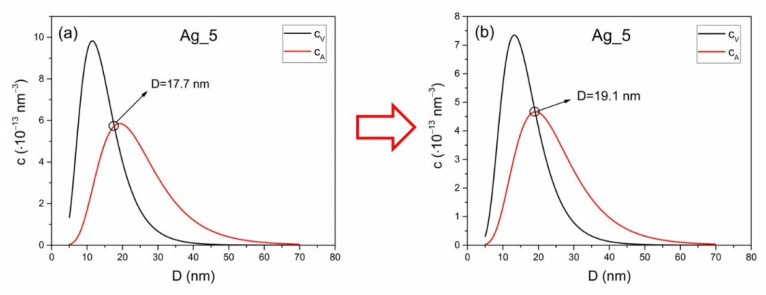
Effect of redshift function on volume concentration in reference to absorbance concentration for sample Ag_5. Notice the shift in intersection diameter from volume average 〈D〉 (**a**) to mode $D_{m}$ (**b**).

Leading to

(13) $$c_{A}=\frac{6V_{c}}{V_{\mathrm{liq}}\pi }\cdot \frac{\int_{D_{\min}}^{D_{\max}}\frac{1}{D^{3}}p\left(D\right)\cdot r\left(D,\lambda \right)\mathrm{dD}}{\int_{D_{\min}}^{D_{\max}}p\left(D\right)\mathrm{dD}}=\frac{6V_{c}}{V_{\mathrm{liq}}\pi }\cdot K\frac{1}{D_{m}}\frac{1}{\left({\left(\Delta \lambda \right)}^{2}+D_{m}^{2}\right)}$$

where $D_{m}$ is the mode diameter of nanoparticles in a colloidal solution. Here, it must be stated that the integral in numerator (13) cannot be analytically solved, so the final analytical form is the result of numerical testing. In Figure 4a, volume and absorbance concentrations as a function of diameter are given. Concentrations are calculated without use of redshift function using (3b) for volume concentration and (9) for absorbance concentration with respect to (10) for conservation of total concentration. In Figure 4b, volume and absorbance concentrations as a function of a diameter are given with use of redshift function. Relation (3b) was used for volume concentration and (3e) for absorbance concentration with respect to (10) for conservation of total concentration. It can be seen that intersection of $c_{A}\left(D\right)$ and $c_{V}\left(D\right)$ differ in Figure 4a,b. The diameter when $c_{A}\left(D\right)=c_{V}\left(D\right)$ is shifted from volume average 〈D〉 to mode $D_{m}$ diameter when redshift function is applied.

With further analysis, through comparison of (11) with (13), one can derive another expression for K:

(14) $$K=e^{\frac{3}{2}s^{2}}\cdot {\left(\frac{\lambda_{\mathrm{SPR}}}{\lambda_{0}}\right)}^{3}$$

Now all parameters of the fitting function are defined, and its initial form (15a) based on the Beer–Lambert law and present form (15b) can be written:

(15a) $$A\left(\lambda \right)=\frac{1}{\ln\left(10\right)}\cdot \sigma_{e}\left(D,\lambda \right)\cdot c\left(D\right)\cdot l+A_{0}$$

(15b) $$A\left(x\right)=\frac{1}{\ln\left(10\right)}\cdot \left(\frac{6V_{c}}{\pi \sqrt{2\pi }{\mathrm{sV}}_{\mathrm{liq}}D_{m}}\cdot e^{-\frac{s^{2}}{2}}\cdot \left(\frac{K_{1}}{x}+\frac{K_{2}}{x^{4}}\cdot {\left(D\frac{x_{0}}{x}\right)}^{3}\right)\cdot e^{-}\frac{{\left(\ln\left(\frac{D\frac{x_{0}}{x}}{D_{m}}\right)\right)}^{2}}{2s^{2}}\cdot l\cdot K\cdot \frac{{\left(D\frac{x}{x_{0}}\right)}^{2}}{{\left(x-x_{0}\right)}^{2}+{\left(D\frac{x}{x_{0}}\right)}^{2}}\right)+A_{0}$$

where x stands for modeled wavelength and $x_{0}$ for modeled resonance wavelength. In the form of the fitting function given by (15b) with initially defined volume of crater $V_{c}$, volume of liquid $V_{\mathrm{liq}}$ and optical path length l, all fitted parameters behave well except constants $K_{1}$ and $K_{2}$, which correspond to dipole absorption and scattering, respectively. Therefore, an attempt is made to increase the interrelation of these parameters in the function. In order to have all fitted parameters of the fitting function well defined in terms of mean value and corresponding error, interrelation between $K_{1}$ and $K_{2}$ is found by equalizing expressions (8) and (14) for K giving:

(16) $$K_{2}=K_{1}\cdot \frac{{\left(e^{\frac{3}{2}s^{2}}{\left(\frac{x_{0}}{\lambda_{0}}\right)}^{3}-1\right)}^{\frac{3}{2}}}{4{\left(1-\frac{\lambda_{0}}{x_{0}}\right)}^{3}}$$

Furthermore, since $x_{0}$ is expected to be equal to $\lambda_{\mathrm{SPR}}$, it means that $x_{0}=\lambda_{0}+\Delta \lambda$ where Δλ can be further expressed using (7) and (14):

(17) $$\Delta \lambda =D_{m}\cdot \sqrt{{\left(\frac{x_{0}}{\lambda_{0}}\right)}^{3}e^{\frac{3}{2}s^{2}}-1}$$

With all the mentioned substitutions, the fitting function in its final form is given by:


(18) $$A\left(x\right)=\frac{1}{\ln\left(10\right)}\cdot (\frac{6{\text{V}}_{\text{c}}}{\pi \sqrt{2\pi }{\mathrm{sV}}_{\mathrm{liq}}D_{m}}\cdot e^{-\frac{s^{2}}{2}}\cdot (\frac{K_{1}}{x}+\frac{K_{1}\cdot \frac{{\left(e^{\frac{3}{2}s^{2}}{\left(\frac{x_{0}}{\lambda_{0}}\right)}^{3}-1\right)}^{\frac{3}{2}}}{4{\left(1-\frac{\lambda_{0}}{x_{0}}\right)}^{3}}}{x^{4}}\cdot {\left(D\frac{x_{0}}{x}\right)}^{3})\cdot e^{-}\frac{{\left(\ln\left(\frac{D\frac{x_{0}}{x}}{D_{m}}\right)\right)}^{2}}{2s^{2}}\cdot l\cdot e^{\frac{3}{2}s^{2}}\cdot {\left(\frac{x_{0}}{\lambda_{0}}\right)}^{3}\cdot \frac{{\left(D\frac{x}{x_{0}}\right)}^{2}}{{\left(x-\left(\lambda_{0}+D_{m}\cdot \sqrt{{\left(\frac{x_{0}}{\lambda_{0}}\right)}^{3}e^{\frac{3}{2}s^{2}}-1}\right)\right)}^{2}+{\left(D\frac{x}{x_{0}}\right)}^{2}})+A_{0}$$

Although complicated, the advantage of this form of fitting function is interrelation of parameters, which are all physical, making them precisely determined. This form of fitting function has been checked on 33 different UV-Vis spectra of silver nanoparticles with results consistent with TEM measurements in the diameter range 20–90 nm. The developed fitting function was checked on the UV-Vis spectra of five colloidal solutions of silver nanoparticles obtained by laser ablation in water done by the authors, 25 UV-Vis spectra of chemically synthesized silver nanoparticles measured by NanoComposix, and three UV-Vis spectra of silver nanoparticles from various authors for all of which the total volume of nanoparticles was known. In Table 1, properties of selected representative types of colloidal silver nanoparticles are presented. Information about the properties of all silver nanoparticles analyzed in this work are provided in Table S1 in Supplementary Materials.

As can be seen from Table 1, fitted diameters are in very good agreement with ones measured by electron microscopy. As can be seen in Table S1 in Supplementary Materials, deviations start to arise for diameters smaller than 20 nm and larger than 90 nm, which is expected due to the fact that Mie theory in dipole limit cannot explain new phenomena in UV-Vis spectra above 90 nm while quantum confinement plays a role when the size of nanoparticles becomes significantly smaller than electron mean free path (~50 nm for Ag) [29]. Therefore, the developed fitting function has a range of applicability, which is 20–90 nm. In Table 2, examples of colloidal silver nanoparticles where the fitting function does not reproduce measured values are shown.

**Figure 5:**
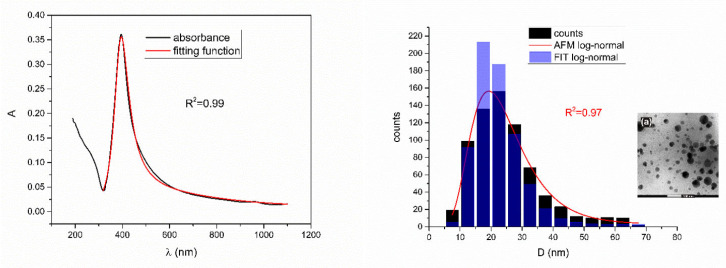
UV-Vis spectrum with fitting function (**left**) for sample Ag_5. Reported and reconstructed size distributions are shown on the (**right**). An additional TEM image is also shown.

**Figure 6:**
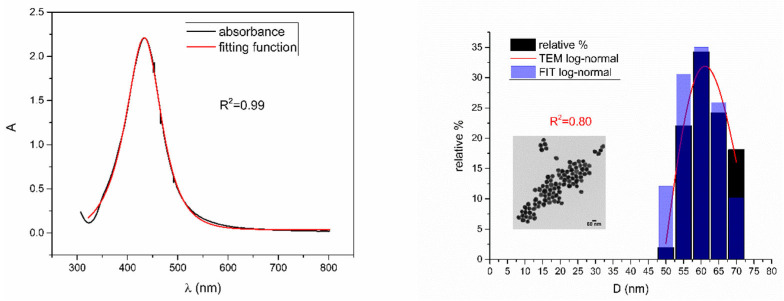
UV-Vis spectrum with fitting function (**left**) for sample NanoX_6. Reported and reconstructed size distributions are shown on the (**right**). An additional TEM image is also shown.

**Figure 7:**
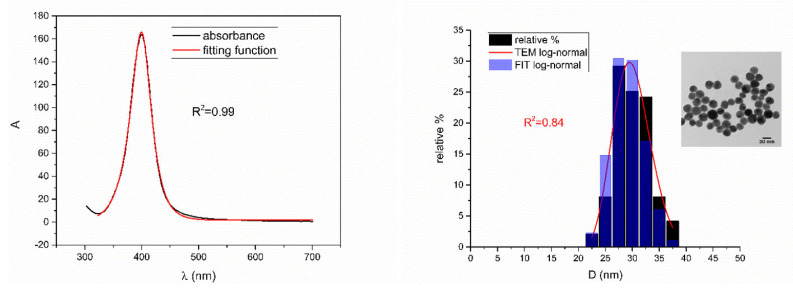
UV-Vis spectrum with fitting function (**left**) for sample BioP_4. Reported and reconstructed size distributions are shown on the (**right**). An additional TEM image is also shown.

**Figure 8:**
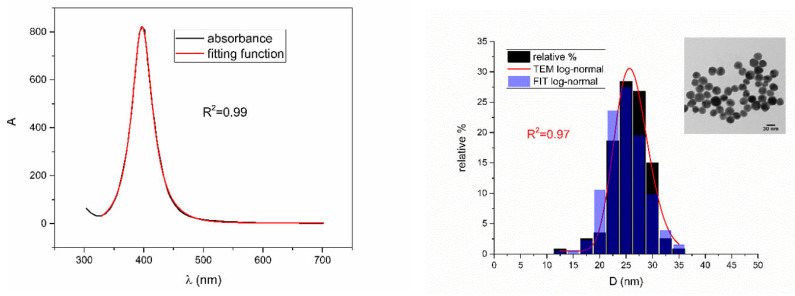
UV-Vis spectrum with fitting function (**left**) for sample EcoX_2. Reported and reconstructed size distributions are shown on the (**right**). An additional TEM image is also shown.

**Figure 9:**
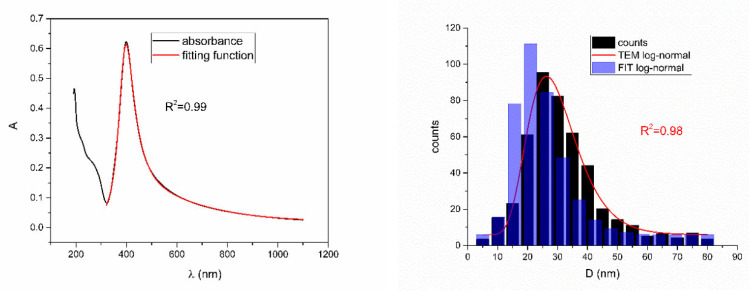
UV-Vis spectrum with fitting function (**left**) for sample AgIn_1. Reported and reconstructed size distributions are shown on the (**right**).

Figure 5, Figure 6, Figure 7, Figure 8 and Figure 9 show UV-Vis spectra, TEM images, as well as measured and reconstructed size distributions of representative colloidal silver nanoparticles selected from each sample group. Table S2 in the Supplementary Materials shows figures of UV-Vis spectra with fit obtained by developed fitting function, TEM images, and associated reported size distributions with their reconstruction from fitted parameters for all samples.

**Figure 10:**
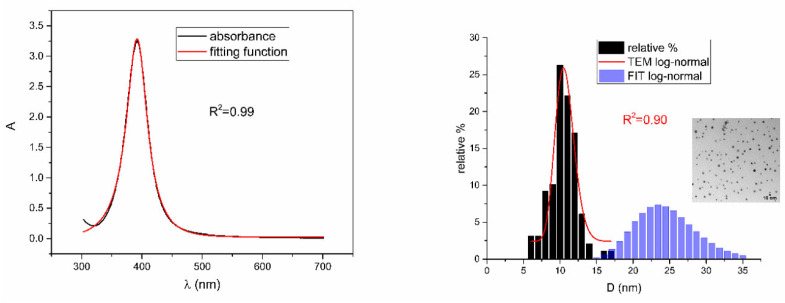
UV-Vis spectrum with fitting function (**left**) for sample NanoX_1. Reported and reconstructed size distributions are shown on the (**right**). An additional TEM image is also shown.

**Figure 11:**
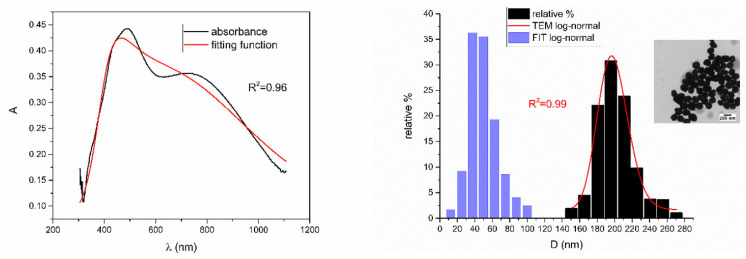
UV-Vis spectrum with fitting function (**left**) for sample NanoX_10. Reported and reconstructed size distributions are shown on the (**right**). An additional TEM image is also shown.

**Figure 12:**
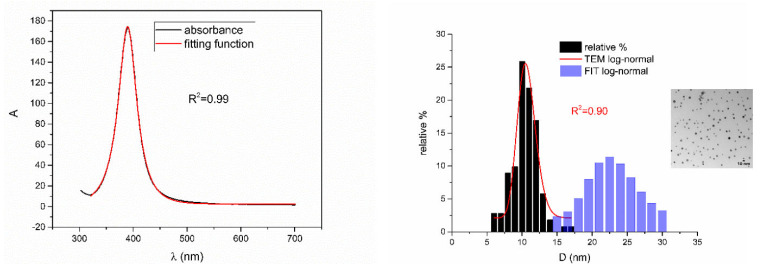
UV-Vis spectrum with fitting function (**left**) for sample BioP_2. Reported and reconstructed size distributions are shown on the (**right**). An additional TEM image is also shown.

**Figure 13:**
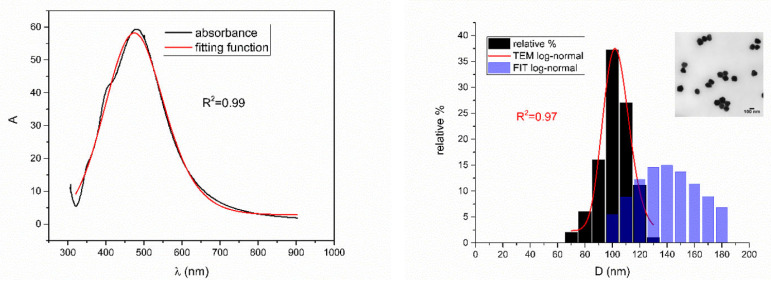
UV-Vis spectrum with fitting function (**left**) for sample BioP_10. Reported and reconstructed size distributions are shown on the (**right**). An additional TEM image is also shown.

**Figure 14:**
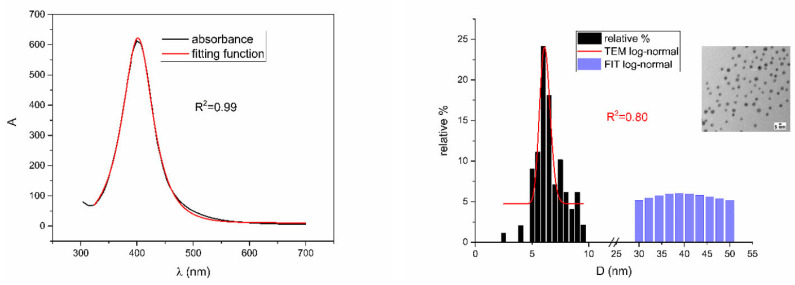
UV-Vis spectrum with fitting function (**left**) for sample EcoX_1. Reported and reconstructed size distributions are shown on the (**right**). An additional TEM image is also shown.

According to Table 2, Figure 10, Figure 11, Figure 12, Figure 13 and Figure 14 are showing examples of colloidal silver nanoparticles where output parameters of fitting function showed a big discrepancy from reported ones.

**Figure 15:**
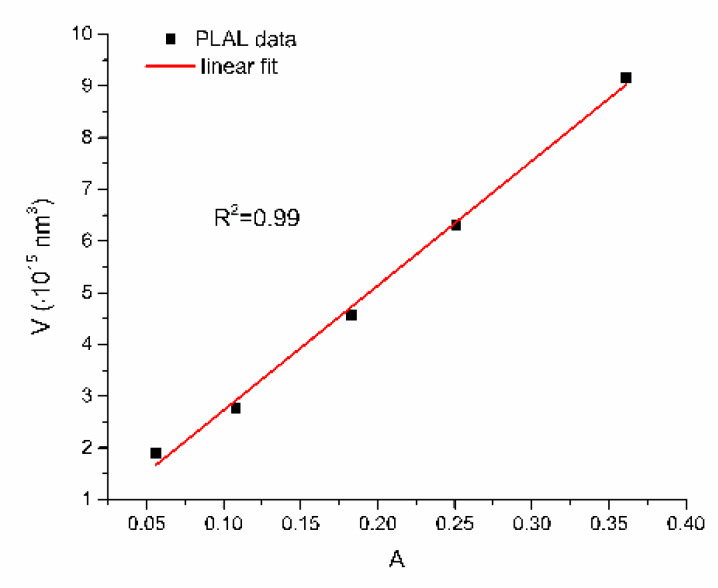
Dependence of crater volume on peak SPR absorbance for PLAL data obtained by the authors. With permission from Springer from [20].

From Figure 5, Figure 6, Figure 7, Figure 8 and Figure 9, where nanoparticles fit the 20–90 nm range, it can be seen that UV-Vis fitting function reproduces the measured ones (with $R^{2}\geq 0.99$). Reconstructed size distributions also represent the measured ones well. In Figure 10, Figure 11, Figure 12, Figure 13 and Figure 14, where nanoparticles are out of the 20–90 nm range, UV-Vis fitting function reproduces the measured ones while reconstructed size distributions fail to reproduce the measured ones. In Table S2 in Supplementary Materials, fitting deviations occur for nanoparticles’ diameters larger than 90 nm where quadrupole terms of extinction cross section appear. Notice that, although for diameters smaller than 20 nm, the fitting function works well, fitting parameters like mode diameter $D_{\mathrm{FIT}}$ do not coincide with measured diameters $D_{\mathrm{TEM}}$. The explanation lies in quantum confinement, which determines the optical behavior of nanoparticles of corresponding sizes. However, the following problem arises: since the diameter and consequently the concentration of nanoparticles in the colloidal solution are not known before measurements and the purpose of the fitting function is to avoid the use of experimental techniques for their determination, certain criteria to the output values of the fitting function should be implied. The reason is in the difference between real and fitted values of nanoparticles’ diameter although the fitting function works well. So, the question is how to, for given UV-Vis spectra, accurately determine the diameter of nanoparticles if the fitting function works well but the real diameters differ from the fitted ones? As can be seen in detail from Tables S1–S3 in Supplementary Materials, the fitting function both fits well and gives accurate diameters as measured ones for the diameter range 20–90 nm but a problem arises outside that range. Since for diameters larger than 90 nm, the fitting function does not work as well, it is logical that output diameters would differ significantly from measured ones. However, for diameters smaller than 20 nm, the fitting function works well but output diameters differ significantly from real ones. In order to deduce if the output values of the fitting function are correct, a column of expected diameters is added to each Table, which was obtained by the above introduced relation $D\left(\lambda \right)=13+0.91\cdot \left|\lambda_{\mathrm{SPR}}-\lambda_{0}\right|$ shown in Figure 3. By comparison of fitted and expected diameters in Table 1 and Table 2, as well as in detailed Table S1 in Supplementary Materials, it can be seen that they almost coincide, meaning that the fitting function outputs diameters that are expected. However, measured diameters are significantly different for sizes smaller than 20 nm. That can be interpreted in two ways: either the reported measured diameters are the subject of error or different criteria must be established. Notice that for the same nanoparticles’ diameters (9.9 nm and 19.9 nm), different SPR wavelengths are reported, which may be the subject of measurement error. The criteria for reliability of fitted parameters is, therefore, the following: (1) uncertainties of each of the nine fitting parameters should be significantly less than their mean fitted value and (2) based on data used in this work, for SPR wavelengths less than 395 nm, the fitting function does not output correct values. The importance of a Lorentzian-like term in the fitting function must be emphasized. It represents a redshift correction to extinction cross section and an attempt to explain its origin. Although a specific functional form was hypothesized, it has been shown that it describes real UV-Vis spectra accurately. The physical explanation can be the following: since localized surface plasmon resonance phenomenon occurs due to collective oscillations of electrons on the boundary between media with opposite signs of dielectric functions under the influence of an oscillating electromagnetic field, the differential equation of the harmonic oscillator with a damping term and driving force can be used to describe it. Since the decaying amplitude of plasmons in frequency space means that oscillation does not consist of a single monochromatic frequency, it can be represented as a sum of monochromatic frequencies with belonging amplitudes. The Fourier transform yields amplitudes of given frequencies with accompanying intensities, which have a Lorentzian functional form. It seems that exactly this Lorentzian form is the origin of redshift correction to extinction cross section derived in the Mie theory and used in Beer–Lambert’s law. Another compelling reason is the fact that scattering cross section in dipole terms for weakly dissipating nanoparticles near SPR with use of Drude permittivity model has Lorentzian functional form [30]. Use of Lorentzian function for redshift accounts mostly for radiative damping term due to increasing sizes of nanoparticles, which is why it works well for silver nanoparticles. More precisely, Grigorchuk [31] showed that for metallic nanoparticles in dielectric medium, radiative decay rate grows quadratically with nanoparticle size, which is accounted for in Lorentzian function in this work. For nanoparticles’ size range with pronounced non-radiative dissipation terms, additional terms should be added to account for electron relaxation processes. Due to damping frequency for non-radiative losses by interband transitions higher than SPR frequency, silver nanoparticles possess superior plasmonic properties compared to other noble metals, such as gold [17]. This fact is visible on UV-Vis spectra of silver nanoparticles in form of one peak due to plasmon band only without interference with interband transitions. Evanoff and Chumanov [32] showed for spherical silver nanoparticles that absorption and scattering contribution to overall extinction are almost equal for nanoparticle size of 52 nm. Therefore, for sizes less than 52 nm absorption term prevails while for sizes above scattering term prevails. Furthermore, since parameters, crater volume $V_{k}$ and SPR wavelength $x_{0}$, should be properly initialized, the question of how to determine them without additional measurements arises. For SPR wavelength, it is easy since it can be in initialized by direct readout at the peak of measured UV-Vis spectra. For crater volume, it is more difficult, especially in the case of chemically synthesized nanoparticles, since they did not originate from any kind of crater volume as is the case for laser ablation. However, it is stated that the crater volume that corresponds to chemically synthesized nanoparticles should correspond to the total mass of synthesized nanoparticles divided by the density of the given material. The question that remains, however, is how to determine it. Since only order of magnitude is crucial for the fitting function to work and since the crater volume represents the amplitude of concentration according to (3b), the following proportionality can be written: $A\sim c\sim V_{k}$. This means that a linear correlation can be found between peak absorbances of UV-Vis spectra and crater volumes. Fitting the linear function of form $V\left(A\right)=a+b\cdot A$ on five samples of laser synthesized nanoparticles made by the authors, a calibration of the crater volume on peak absorbance can be made. It must be emphasized that this linear dependence is under the assumption that the diameter of nanoparticles remains constant for all crater volumes and corresponding peak absorbances of UV-Vis spectra. This assumption not only allows this kind of calibration but is also reasonable because it means that the parameter of the mode diameter in the fitting function should always be initialized at the same value. Since the diameters of nanoparticles in the colloidal solutions of these five samples are ca 20 nm, it is assumed that this value is initial before using the fitting function. Linear function performed on these five data pairs gave the following dependence: $V\left(A\right)=3.1\cdot 10^{14}+2.4\cdot 10^{16}\cdot A$ in units of nm^3^. Therefore, using this relation, one can estimate the order of magnitude of crater volume corresponding to the total volume of nanoparticles in the colloidal solution. The linear graph of $V\left(A\right)$ is shown in Figure 15.

In Table 3 are given reported concentration, fitted concentration without redshift function, and fitted concentration with redshift function for comparison. Table 3 contains data for representative colloidal silver nanoparticles selected from each sample group while Table S3 in the Supplementary Materials contains data for all samples.

In Table 4 are given the same examples as in Table 2, for which discrepancy between reported and fitted concentrations exist.

**Figure 16:**
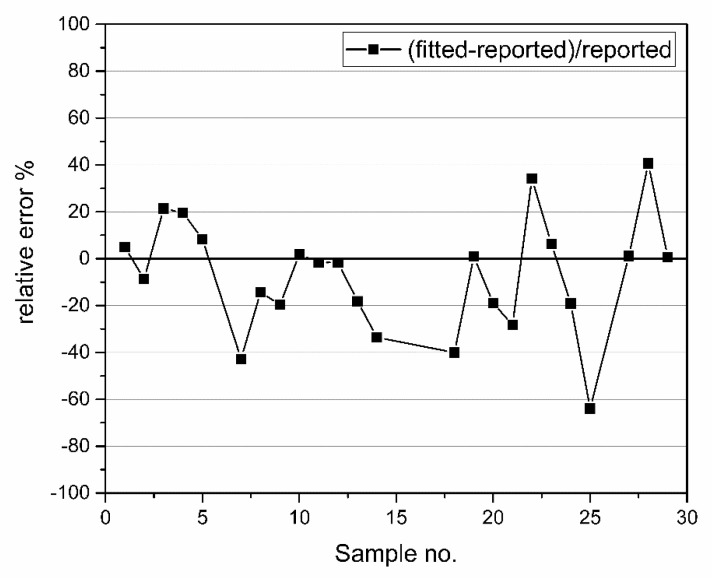
Comparison between relative error of fitted to reported concentration for the set of samples given in Table S3.

Relative errors of fitted to reported concentrations are shown in Figure 16. Notice that samples NanoX_1, NanoX_10, BioP_1, BioP_2, EcoX_1, and EcoX_5 are not shown because their relative error surpasses reasonable range. Those are samples for which the fitting function does not give accurate parameters and correspond to previously mentioned diameter ranges smaller than 20 and larger than 90 nm. Moreover, for AgIn_1, only estimate of order of magnitude of concentration is reported. Figure 16 shows comparison between calculated relative error of fitted to reported concentration for every sample in Table S3 in Supplementary Materials, except those which are outside the 20–90 nm diameter range. Notice that higher discrepancy between fitted and reported values appears for nanoparticles with coated surface for their stabilization in colloidal solutions (Econix, PVP) and those outside the 20–90 nm range. Moreover, in Figure 16, relative errors are mostly negative, meaning that the fitting function underestimates reported number concentrations. A possible reason is that the reported nanoparticles number concentration was determined using mass concentration of silver obtained by ICP-MS, which was then converted by assuming that average nanoparticle mass is the product of bulk silver density and volume per nanoparticle. Discrepancy occurs due to fact that bulk and nanoparticle density usually differ as well due to use of the volume of nanoparticles’ average diameter obtained by TEM. As expected, redshift function has no effect on the value of the total concentration of nanoparticles in a colloidal solutions.

**Figure 17:**
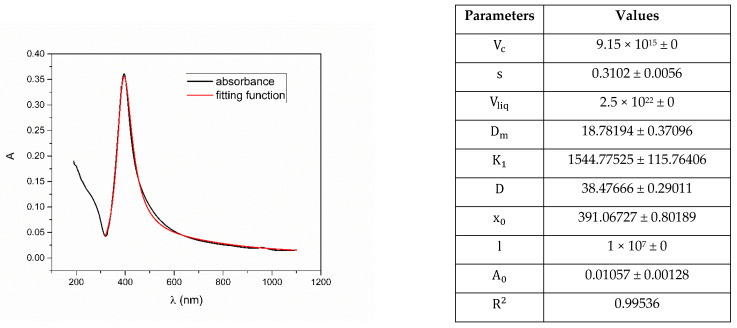
Example of fitting function on a colloidal solution of nanoparticles Ag_5 obtained by the authors with a complete set of fitted parameters. Notice small standard deviations of each parameter and high $R^{2}$ value.

Example of a complete set of fitted parameters for sample Ag_5 is given in Figure 17. As it can be seen, only three parameters out of nine are initially fixed (crater volume $V_{c}$, volume of liquid $V_{\mathrm{liq}}$, and optical path length l) according to experimental setup values, while others are initialized although not fixed and are the result of fitting. Notice, there are small corresponding errors for each fitting parameter as a result of their mutual dependency and interrelation in definition of fitting function.

## 3. Conclusions

In this work, we presented completely derived fitting procedure for UV-Vis spectra of silver nanoparticles in colloidal solutions based on the Beer–Lambert law and Mie theory. All parameters in fitting function are physical, which makes it useful for determining the size distribution of nanoparticles as well as their concentration in solutions. The range of applicability of the fitting function is a nanoparticle diameter of 20–90 nm, for which it gives reliable results. For diameters below 20 nm, the fitting function behaves well although it gives inaccurate mode diameters of nanoparticles, which means that quantum confinement must be taken into consideration due to comparability of electron mean free path and size of nanoparticles. On the other hand, for diameters above 90 nm, due to quadrupole and octupole terms emerging in extinction cross sections, the fitting function is not as good as for lower diameter values since it is based on dipole terms only. It must be emphasized that on the mentioned number of samples, the fitting function in applicability range works well independent of synthesis techniques of nanoparticles—both chemically and laser synthesized. In order to validate the obtained fitted parameters, size distribution reconstruction was done and compared with reported histograms of relative % vs. diameter of nanoparticles by NanoComposix (San Diego, CA, USA). Reconstructed size distributions show good coincidence with reported ones by TEM measurements, which approves an existing form of fitting function. Moreover, we introduced an additional crucial term for redshift correction of extinction cross section that has a Lorentzian-like shape and is responsible for well-defined behavior of the fitting function. Its origin is elaborated through harmonic oscillator behavior of collective oscillations of electrons on localized plasmon resonance wavelength. Since the solution of the differential equation of harmonic oscillator in its Fourier transform is Lorentzian, which describes the shape of broadening linewidth, by analogy we state that redshift correction Lorentzian-like term has the same roots. An additional reason is the fact that for weakly dissipating nanoparticles near SPR, scattering cross section with Drude permittivity model has a Lorentzian functional form. This fitting function has few requirements though: parameters for the volume of crater and SPR wavelength should be properly initialized. It means that for both chemically and laser synthesized nanoparticles, the order of magnitude of the crater volume (or total mass of nanoparticle material) should be given. The main advantage of the developed fitting function is its analytical form and interrelation between parameters, resulting in their high precision after fitting. Future prospects include widening the range of applicability of the fitting function by including quadrupole term and quantum-mechanical effects in dielectric function of nanoparticles’ material as well as the problem of multiple scattering occurring for highly concentrated colloidal solutions.

## Figures and Tables

**Table 1:** List of labels, types, SPR wavelengths, measured, fitted, and expected diameters of representative colloidal silver nanoparticles selected from each sample group with references.

Sample Number	Label	Type	SPR Wavelength	Measured Diameter (nm)	Fitted Diameter (nm)	Expected Diameter (nm)	Ref.
5	Ag_5	PLAL, 5000 p	395	(19.3 ± 0.1)	(18.9 ± 0.4)	(23.0 ± 2.1)	[20]
11	NanoX_6	NanoXact	430	(59 ± 5)	(58.9 ± 0.4)	(54.9 ± 2.7)	[22]
19	BioP_4	BioPure	400	(28.8 ± 3.2)	(28.7 ± 0.2)	(27.6 ± 2.1)	[23]
27	EcoX_2	Econix	397	(24 ± 3)	(24.4 ± 0.2)	(24.8 ± 2.1)	[24]
31	AgIn_1	PLAL	400	(26.3 ± 0.4)	(20.7 ± 0.1)	(27.6 ± 2.1)	[25]

**Table 2:** List of labels, types, SPR wavelengths, measured, fitted, and expected diameters of colloidal silver nanoparticles for which the fitting function does not reproduce measured values.

Sample Number	Label	Type	SPR WaveLength	Measured Diameter (nm)	Fitted Diameter (nm)	Expected Diameter (nm)	Ref.
6	NanoX_1	NanoXact	392	(9.9 ± 1.9)	(23.5 ± 0.2)	(20.3 ± 2.1)	[22]
15	NanoX_10	NanoXact	486	(193.7 ± 21.2)	(43 ± 3)	(105.8 ± 4.6)	[22]
17	BioP_2	BioPure	390	(9.9 ± 1.9)	(22.4 ± 0.1)	(18.5 ± 2.0)	[23]
25	BioP_10	BioPure	486	(97 ± 11)	(137 ± 3)	(105.8 ± 4.6)	[23]
26	EcoX_1	Econix	401	(6.3 ± 1.3)	(38.8 ± 0.5)	(28.5 ± 2.1)	[24]

**Table 3:** Labels, types, shapes, reported and fitted concentrations for representative colloidal solutions of silver nanoparticles with corresponding error.

Sample No.	Label	Type	Shape	Reported Concentration (1010 cm^−3^)	Fitted Concentration without $r\left(D,\lambda \right)$ (1010 cm^−3^)	Fitted Concentration with $r\left(D,\lambda \right)$ (1010 cm^−3^)
5	Ag_5	PLAL, 5000 p	spherical	10.4	(11.9 ± 0.7)	(10.4 ± 0.7)
11	NanoX_6	NanoXact	spherical	1.9	(1.96 ± 0.04)	(1.87 ± 0.04)
19	BioP_4	BioPure	spherical	770	(790 ± 20)	(780 ± 20)
27	EcoX_2	Econix	spherical	6500	(6800 ± 200)	(6600 ± 200)
31	AgIn_1	PLAL	spherical	1	(23.9 ± 0.3)	(20.5 ± 0.3)

**Table 4:** Labels, types, shapes, reported and fitted concentrations for the same examples from Table 2 where the fitting function does not work.

Sample No.	Label	Type	Shape	Reported Concentration (1010 cm^−3^)	Fitted Concentration without $r\left(D,\lambda \right)$ (1010 cm^−3^)	Fitted Concentration with $r\left(D,\lambda \right)$ (1010 cm^−3^)
6	NanoX_1	NanoXact	spherical	370	(29.2 ± 0.7)	(28.0 ± 0.7)
15	NanoX_10	NanoXact	spherical	0.055	(6 ± 1)	(5 ± 1)
17	BioP_2	BioPure	spherical	20,000	(1810 ± 20)	(1750 ± 20)
25	BioP_10	BioPure	spherical	21	(8.0 ± 0.5)	(7.6 ± 0.5)
26	EcoX_1	Econix	spherical	370,000	(1640 ± 60)	(1570 ± 60)

## Data Availability

Not applicable.

## Supplementary Materials

The following supporting information can be downloaded at https://www.mdpi.com/article/10.3390/nano12193302/s1, Table S1: Complete list of labels, types, shapes, measured and fitted diameters of colloidal silver nanoparticles for 33 different samples with references, Table S2: Analyzed samples with fit of UV-Vis spectra and reconstruction of size distribution obtained by Formulae (18) and (3d) respectively. TEM images are provided for NanoComposix samples, Table S3: Labels, types, shapes, reported and fitted concentrations for colloidal solutions of nanoparticles with corresponding error; S4: Procedure for size distribution reconstruction.

## Code Availability

Code generated by programming with given equations is available from the corresponding author on reasonable request.

## Table of Parameters (Abbreviations)

SPR	surface plasmon resonance
A	absorbance
$A_{\mathrm{SPR}}$	peak absorbance at SPR
x	modeled wavelength
$x_{0}$	modeled SPR wavelength
λ	wavelength
$\lambda_{0}$	theoretical SPR wavelength
$\lambda_{\mathrm{SPR}}$	experimental SPR wavelength
$V_{c}$	crater volume (total volume of all nanoparticles)
$V_{\mathrm{liq}}$	volume of liquid
s	standard deviation of log-normal size distribution
$s_{\mathrm{TEM}}$	standard deviation of log-normal size distribution obtained by TEM imaging
$s_{\mathrm{FIT}}$	standard deviation of log-normal size distribution obtained by fitting function
$D_{m}$	mode diameter of nanoparticles
$D_{\mathrm{TEM}}$	reported mode diameter of nanoparticles obtained by TEM imaging
$D_{\mathrm{FIT}}$	mode diameter of nanoparticles obtained by fitting function
D	modeled effective diameter
$D_{M}$	simple model diameter
$K_{1}$	effective absorption term of extinction cross section
$K_{10}$	effective absorption term of extinction cross section for $\lambda_{0}$
$K_{1\mathrm{SPR}}$	effective absorption term of extinction cross section for $\lambda_{\mathrm{SPR}}$
$K_{2}$	effective scattering term of extinction cross section
$K_{20}$	effective scattering term of extinction cross section for $\lambda_{0}$
$K_{2\mathrm{SPR}}$	effective scattering term of extinction cross section for $\lambda_{\mathrm{SPR}}$
l	optical path length
$A_{0}$	scaling background absorbance
$I_{t}$	transmitted light intensity
$I_{0}$	incident light intensity
$\sigma_{a}$	absorption cross section
$\sigma_{s}$	scattering cross section
$\sigma_{e}$	extinction cross section
$\sigma_{g}$	geometric cross section
$\varepsilon_{1}$	real part of metal dielectric function
$\varepsilon_{2}$	imaginary part of metal dielectric function
$\varepsilon_{m}$	real part of medium dielectric function
$c_{V}$	volume concentration
$c_{A}$	absorbance concentration
$N_{j}\left(D_{j}\right)$	number of nanoparticles with diameter $D_{j}$
$p\left(D_{j}\right)$	probability of occurrence of diameter $D_{j}$
$f\left(D_{j}\right)$	log-normal distribution function for diameters $D_{j}$
P	integral area under log-normal distribution function
$r\left(D,\lambda \right)$	redshift function
K	non-dimensional redshift constant
Δλ	shift of experimental from theoretical SPR wavelength
$D_{\min}$	diameter corresponding to minimum of extinction to geometric cross section ratio
$D_{0}$	=$D_{\min}$ for fixed $\lambda_{0}$
$D_{\mathrm{SPR}}$	=$D_{\min}$ for fixed $\lambda_{\mathrm{SPR}}$
