# Supplementary material for: Fitting Procedure to Reconstruct the Size Distribution and the Concentration of Silver Colloidal Nanoparticles from UV-Vis Spectra
Source: Nanomaterials (Basel). 2022 Sep 22;12(19):3302. doi: 10.3390/nano12193302 (PMC9565506; doi:10.3390/nano12193302)
Supplement: Supplementary file 1 [file nanomaterials-12-03302-s001.zip › nanomaterials-1904364-supplementary.pdf]

nanomaterials

MDPI

Supplementary Materials

Fitting Procedure to Reconstruct the Size Distribution and the Concentration of Silver Colloidal Nanoparticles from UV-Vis Spectra

Julio Car and Nikša Krstulović *

Institute of Physics, Bijenička cesta 46, 10000 Zagreb, Croatia
* Correspondence: niksak@ifs.hr

Table S1. Complete list of labels, types, shapes, measured and fitted diameters of colloidal silver nanoparticles for 33 different samples with references. For first 4 samples in column measured diameter, simple model diameters from [20] are reported.

Sample Number	Label	Type	SPR Wavelength	Measured Diameter (nm)	Fitted Diameter (nm)	Expected Diameter (nm)	Ref.
1	Ag_1	PLAL, 500 p	397	(19.4±2.2)	(19.7±1.2)	(24.8±2.1)	[20]
2	Ag_2	PLAL, 1000 p	395	(18.3±1.6)	(19.5±0.7)	(23.0±2.1)	[20]
3	Ag_3	PLAL, 2000 p	395	(19.5±1.3)	(18.8±0.4)	(23.0±2.1)	[20]
4	Ag_4	PLAL, 3000 p	395	(19.3±1.0)	(18.7±0.3)	(23.0±2.1)	[20]
5	Ag_5	PLAL, 5000 p	395	(19.3±0.1)	(18.9±0.4)	(23.0±2.1)	[20]
6	NanoX_1	NanoXact	392	(9.9±1.9)	(23.5±0.2)	(20.3±2.1)	[22]
7	NanoX_2	NanoXact	392	(19.9±2.8)	(23.8±0.2)	(20.3±2.1)	[22]
8	NanoX_3	NanoXact	400	(29±3)	(30.3±0.2)	(27.6±2.1)	[22]
9	NanoX_4	NanoXact	414	(41±3)	(43.7±0.3)	(40.3±2.4)	[22]
10	NanoX_5	NanoXact	422	(51±6)	(50.0±0.3)	(47.6±2.5)	[22]
11	NanoX_6	NanoXact	430	(59±5)	(58.9±0.4)	(54.9±2.7)	[22]
12	NanoX_7	NanoXact	449	(73±8)	(74.2±0.7)	(72.2±3.3)	[22]
13	NanoX_8	NanoXact	455	(79±7)	(85±1)	(77.6±3.5)	[22]
14	NanoX_9	NanoXact	485	(95±9)	(108.3±4.8)	(104.9±4.5)	[22]
15	NanoX_10	NanoXact	486	(193.7±21.2)	(43±3)	(105.8±4.6)	[22]
16	BioP_1	BioPure	400	(5.2±0.9)	(39.1±0.1)	(27.6±2.1)	[23]
17	BioP_2	BioPure	390	(9.9±1.9)	(22.4±0.1)	(18.5±2.0)	[23]
18	BioP_3	BioPure	391	(19.9±2.8)	(23.6±0.2)	(19.4±2.0)	[23]
19	BioP_4	BioPure	400	(28.8±3.2)	(28.7±0.2)	(27.6±2.1)	[23]
20	BioP_5	BioPure	412	(39±4)	(42.2±0.3)	(38.5±2.3)	[23]
21	BioP_6	BioPure	424	(48±5)	(53.4±0.4)	(49.4±2.6)	[23]
22	BioP_7	BioPure	426	(59±6)	(53.7±0.3)	(51.2±2.6)	[23]
23	BioP_8	BioPure	441	(70.0±6.6)	(68.6±0.5)	(64.9±3.1)	[23]
24	BioP_9	BioPure	455	(79.1±6.8)	(85±1)	(77.6±3.5)	[23]
25	BioP_10	BioPure	486	(97±11)	(137±3)	(105.8±4.6)	[23]
26	EcoX_1	Econix	401	(6.3±1.3)	(38.8±0.5)	(28.5±2.1)	[24]
27	EcoX_2	Econix	397	(24±3)	(24.4±0.2)	(24.8±2.1)	[24]
28	EcoX_3	Econix	430	(51±9)	(45±2)	(54.9±2.7)	[24]
29	EcoX_4	Econix	465	(77.5±7.0)	(78±2)	(86.7±3.8)	[24]
30	EcoX_5	Econix	503	(106±14)	(76±3)	(121.3±5.2)	[24]

31	AgIn_1	PLAL	400	(26.3±0.4)	(20.7±0.1)	(27.6±2.1)	[25]
32	AgIn_2	reduction of $AgNO_3$	412	(15±5) radius	(30±5)	(39.1±2.3)	[26]
33	AgIn_3	reduction of $AgNO_3$	443	(34±5) radius	(80±6)	(67.4±3.1)	[27]

Table S2. Analyzed samples with fit of UV-Vis spectra and reconstruction of size distribution obtained by formulae (18) and (3d) respectively. TEM images are provided for NanoComposix samples.

Sample	Fit of UV-Vis Spectra	Reconstruction of Size Distribution
Ag_1<br>D_M=(19.4±2.2)<br>D_{FIT}=(19.7±1.2)<br>s_{TEM}=(-)<br>s_{FIT}=(0.35±0.03)	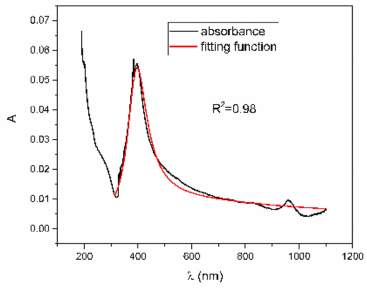	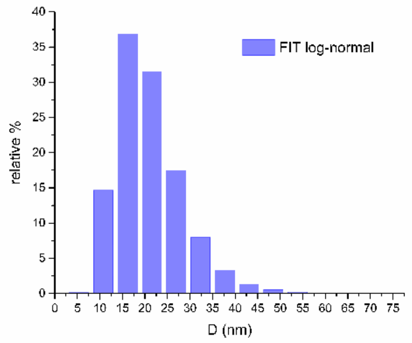
Ag_2<br>D_M=(18.3±1.6)<br>D_{FIT}=(19.5±0.7)<br>s_{TEM}=(-)<br>s_{FIT}=(0.34±0.02)	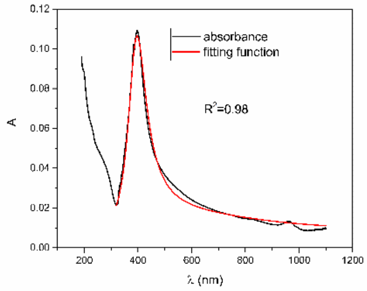	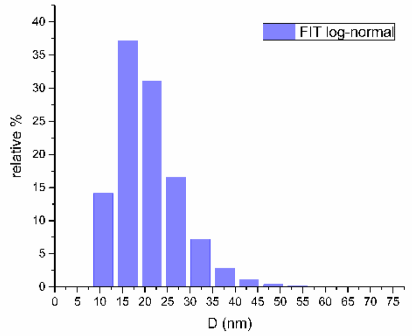
Ag_3<br>D_M=(19.5±1.3)<br>D_{FIT}=(18.8±0.4)<br>s_{TEM}=(-)<br>s_{FIT}=(0.33±0.01)	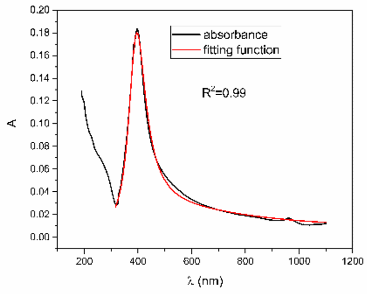	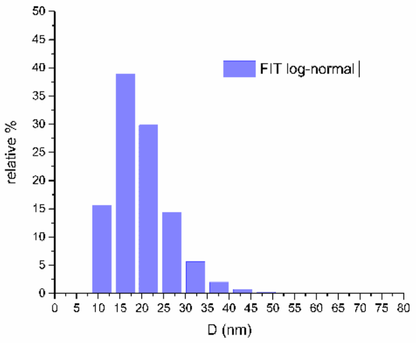

Ag_4

D_{M}=(19.3±1.0)
D_{FIT}=(18.7±0.3)

s_{TEM}=(-)
s_{FIT}=(0.322±0.008)

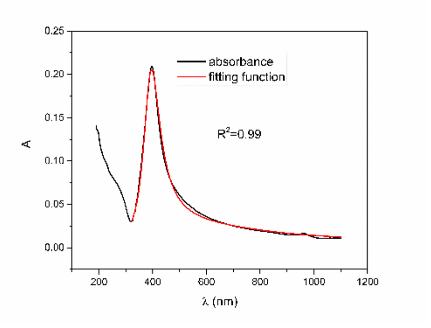

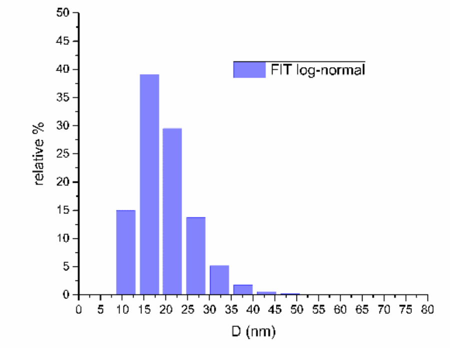

Ag_5

D_{AFM}=(19.3±0.1)
D_{FIT}=(18.9±0.4)

s_{AFM}=(0.41±0.03)
s_{FIT}=(0.309±0.005)

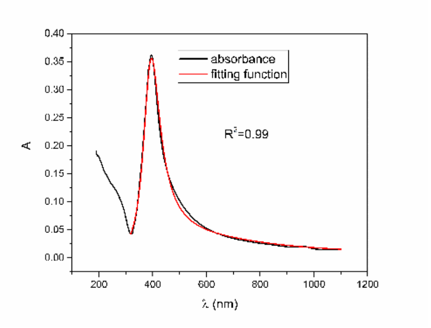

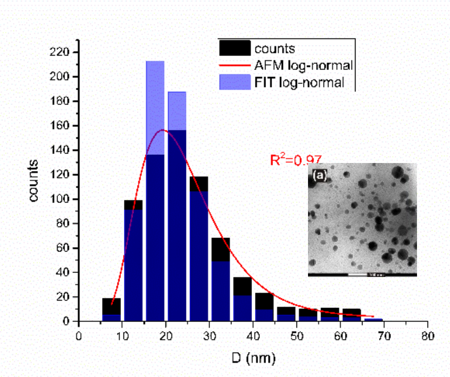

NanoX_1

D_{TEM}=(9.9±1.9)
D_{FIT}=(23.5±0.2)

s_{TEM}=(0.12±0.02)
s_{FIT}=(0.172±0.005)

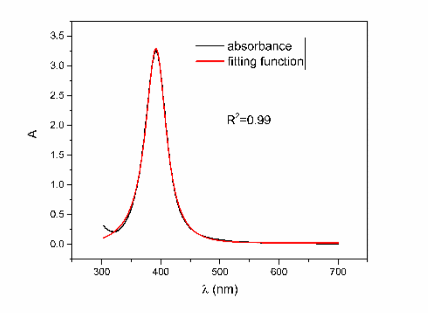

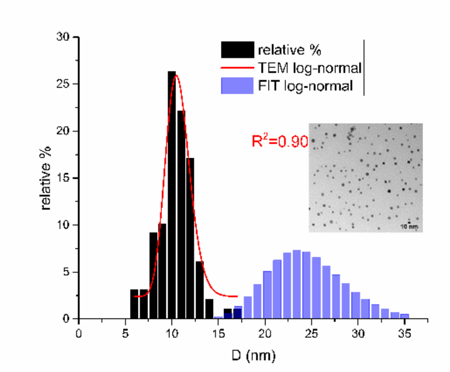

NanoX_2

D_{TEM}=(19.9±2.8)
D_{FIT}=(23.8±0.2)

s_{TEM}=(0.12±0.01)
s_{FIT}=(0.120±0.003)

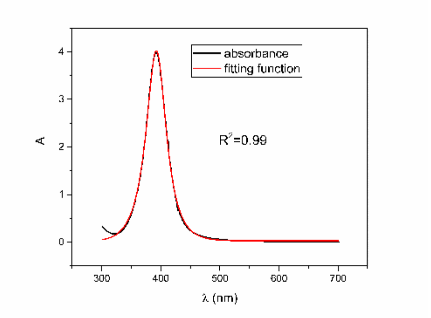

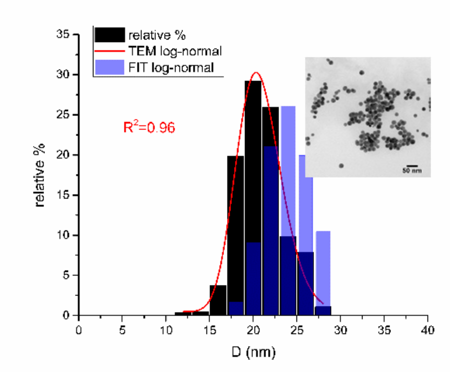

NanoX_3

D_{TEM}=(29±3)
D_{FIT}=(30.3±0.2)

s_{TEM}=(0.12±0.04)
s_{FIT}=(0.110±0.002)

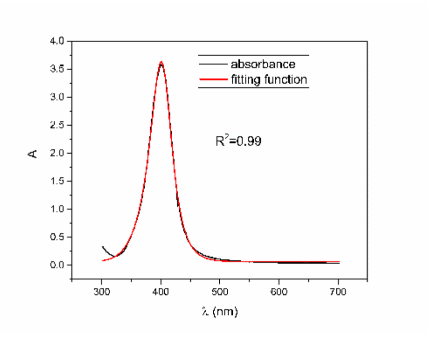

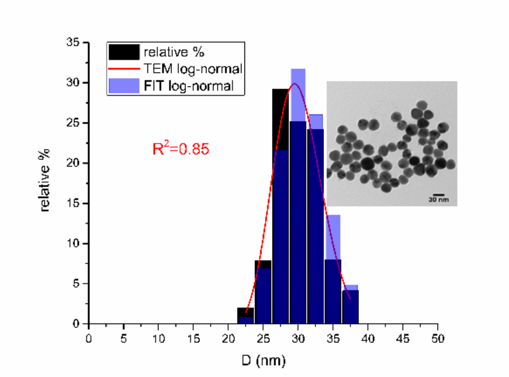

NanoX_4

D_{TEM}=(41±3)
D_{FIT}=(43.7±0.3)

s_{TEM}=(0.12±0.04)
s_{FIT}=(0.121±0.002)

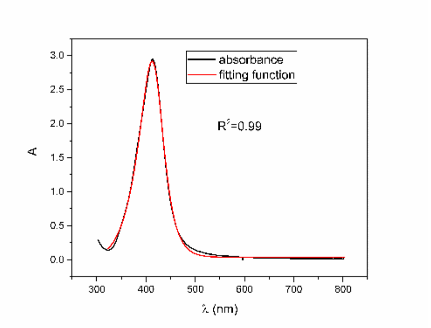

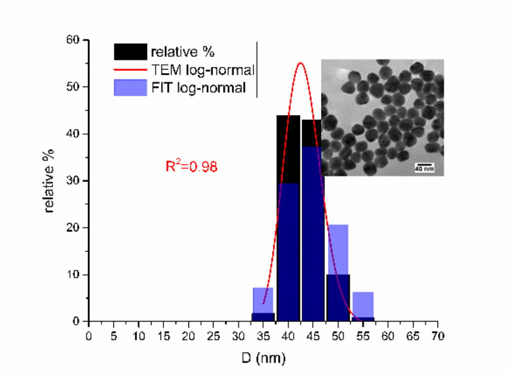

NanoX_5

D_{TEM}=(51±6)
D_{FIT}=(50.0±0.3)

s_{TEM}=(0.119±0.005)
s_{FIT}=(0.153±0.003)

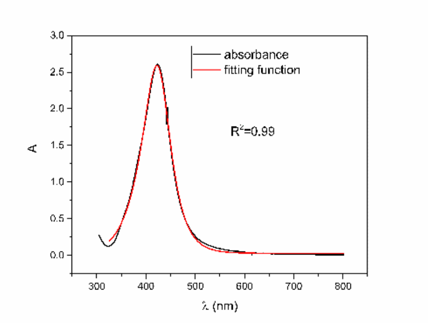

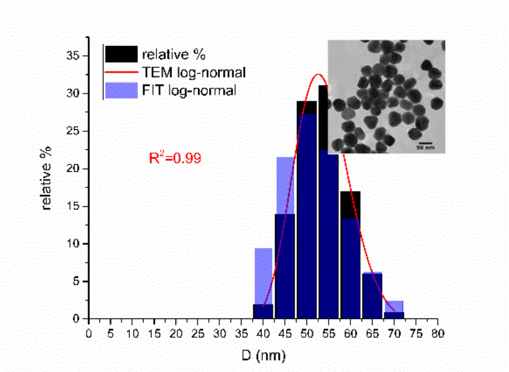

NanoX_6

D_{TEM}=(59±5)
D_{FIT}=(58.9±0.4)

s_{TEM}=(0.18±0.26)
s_{FIT}=(0.178±0.003)

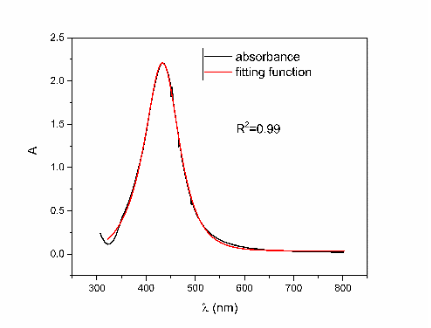

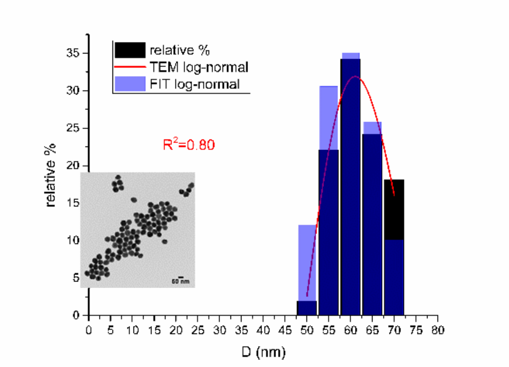

NanoX_7

D_{TEM}=(73±8)
D_{FIT}=(74.2±0.7)

s_{TEM}=(0.08±0.01)
s_{FIT}=(0.221±0.004)

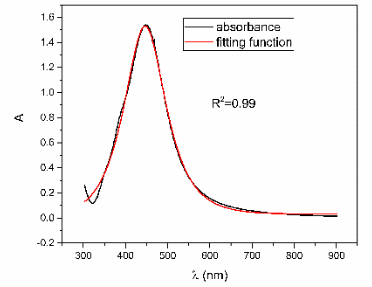

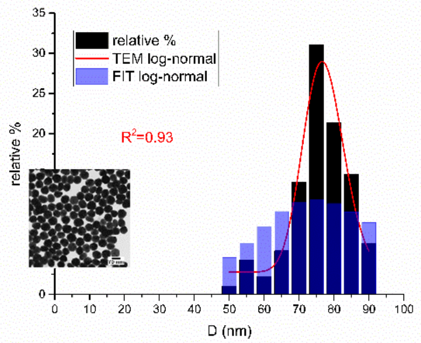

NanoX_8

D_{TEM}=(79±7)
D_{FIT}=(85±1)

s_{TEM}=(0.083±0.007)
s_{FIT}=(0.211±0.004)

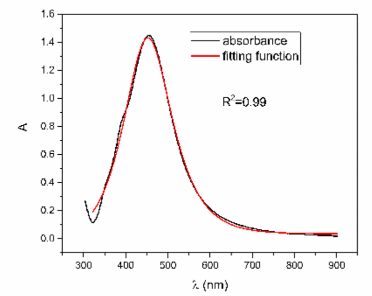

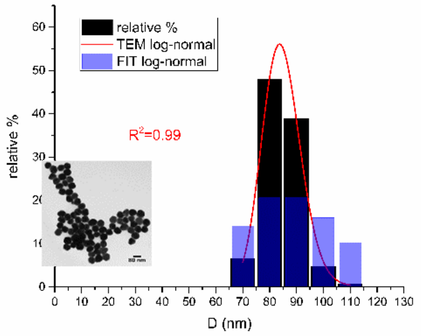

NanoX_9

D_{TEM}=(95±9)
D_{FIT}=(108.3±4.8)

s_{TEM}=(0.092±0.004)
s_{FIT}=(0.159±0.002)

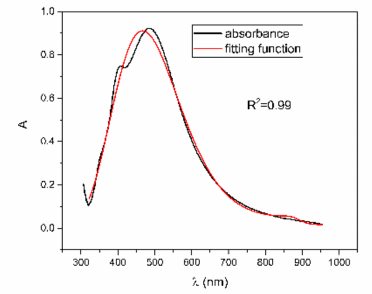

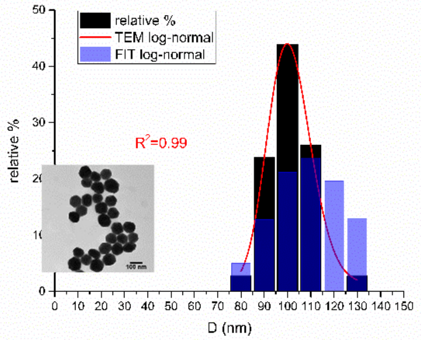

NanoX_10

D_{TEM}=(193.7±21.2)
D_{FIT}=(43±3)

s_{TEM}=(0.092±0.004)
s_{FIT}=(0.30±0.01)

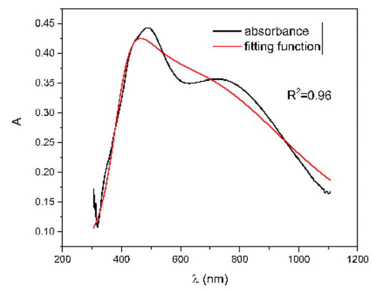

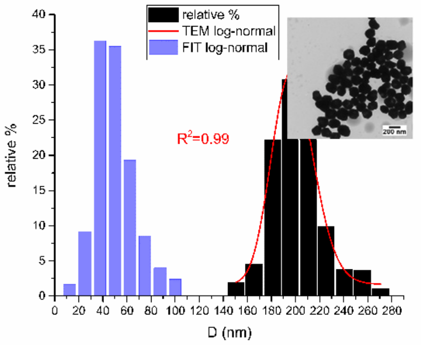

BioP_1

D_{TEM}=(5.2±0.9)
D_{FIT}=(39.1±0.1)

s_{TEM}=(0.13±0.01)
s_{FIT}=(0.201±0.002)

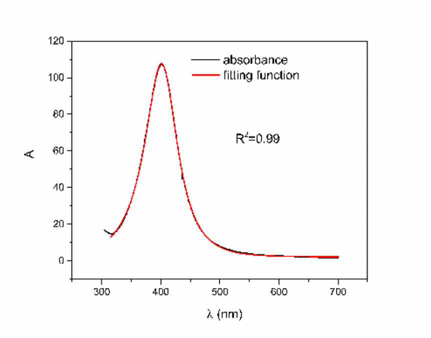

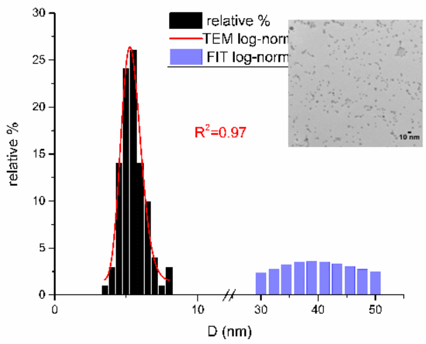

BioP_2

D_{TEM}=(9.9±1.9)
D_{FIT}=(22.4±0.1)

s_{TEM}=(0.12±0.02)
s_{FIT}=(0.144±0.002)

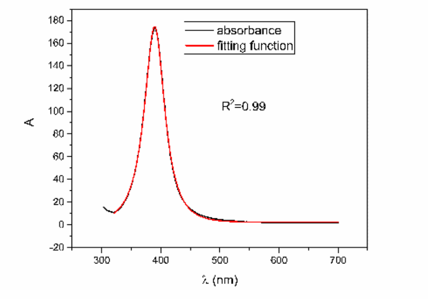

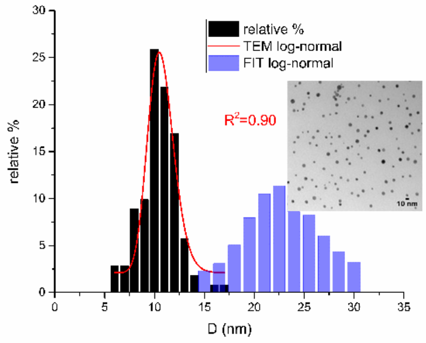

BioP_3

D_{TEM}=(19.9±2.8)
D_{FIT}=(23.6±0.2)

s_{TEM}=(0.12±0.01)
s_{FIT}=(0.110±0.002)

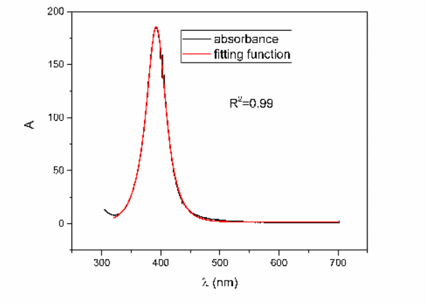

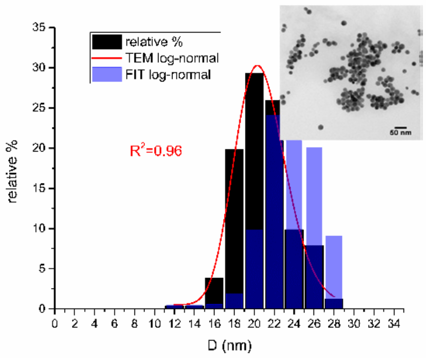

BioP_4

D_{TEM}=(28.8±3.2)
D_{FIT}=(28.7±0.2)

s_{TEM}=(0.12±0.04)
s_{FIT}=(0.111±0.002)

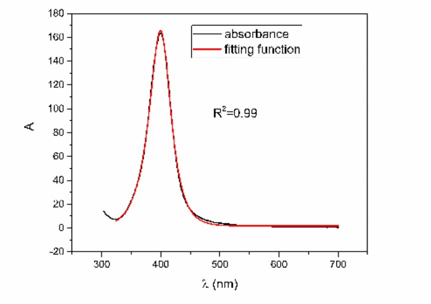

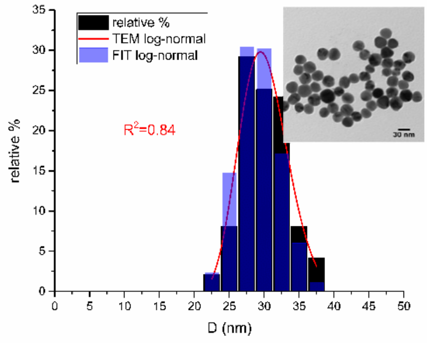

BioP_5<br>D_{TEM}=(39±4)<br>D_{FIT}=(42.2±0.3)<br>s_{TEM}=(0.09±0.01)<br>s_{FIT}=(0.115±0.002)	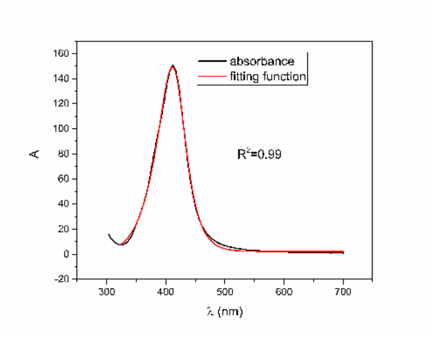	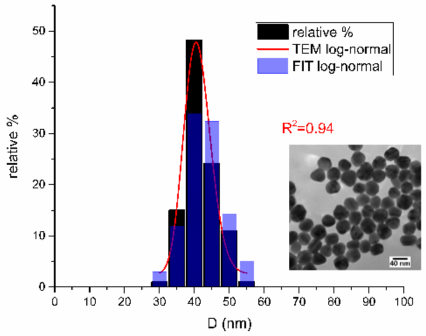
BioP_6<br>D_{TEM}=(48±5)<br>D_{FIT}=(53.4±0.4)<br>s_{TEM}=(0.22±0.22)<br>s_{FIT}=(0.151±0.003)	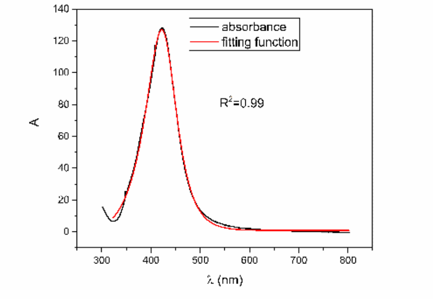	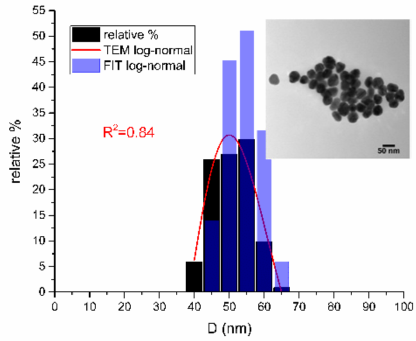
BioP_7<br>D_{TEM}=(59±6)<br>D_{FIT}=(53.7±0.3)<br>s_{TEM}=(0.094±0.007)<br>s_{FIT}=(0.193±0.002)	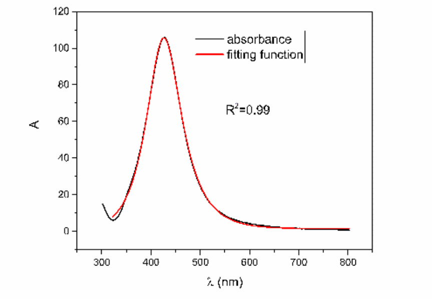	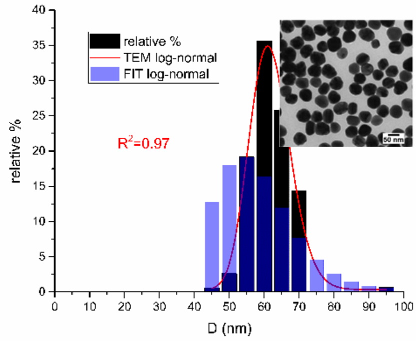
BioP_8<br>D_{TEM}=(70.0±6.6)<br>D_{FIT}=(68.6±0.5)<br>s_{TEM}=(0.03±0.01)<br>s_{FIT}=(0.208±0.003)	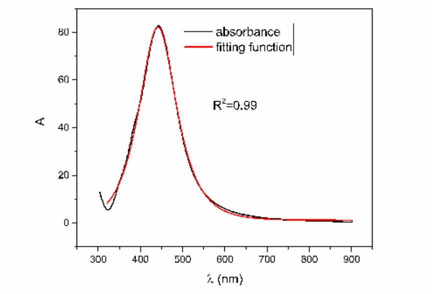	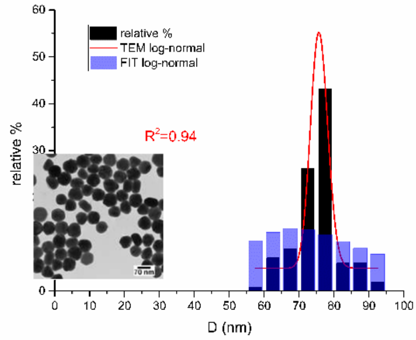

BioP_9<br><br>D_{TEM}=(79.1±6.8)<br>D_{FIT}=(85±1)<br><br>S_{TEM}=(0.083±0.07)<br>S_{FIT}=(0.206±0.004)	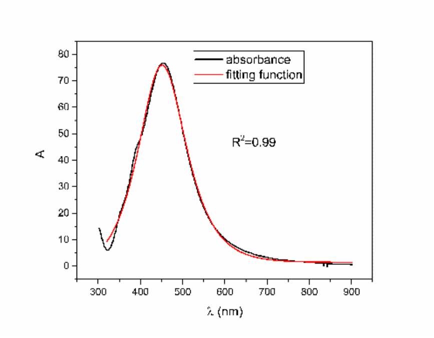	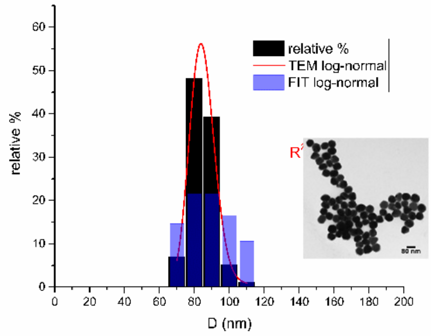
BioP_10<br><br>D_{TEM}=(97±11)<br>D_{FIT}=(137±3)<br><br>S_{TEM}=(0.093±0.008)<br>S_{FIT}=(0.189±0.003)	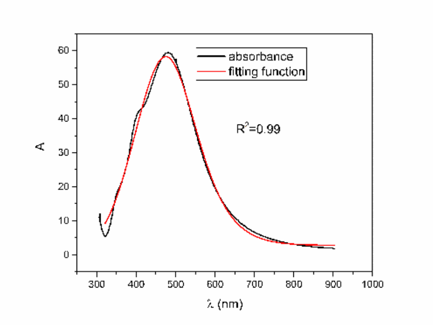	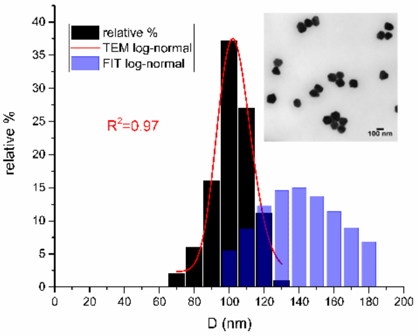
EcoX_1<br><br>D_{TEM}=(6.3±1.3)<br>D_{FIT}=(38.8±0.5)<br><br>S_{TEM}=(0.08±0.01)<br>S_{FIT}=(0.172±0.007)	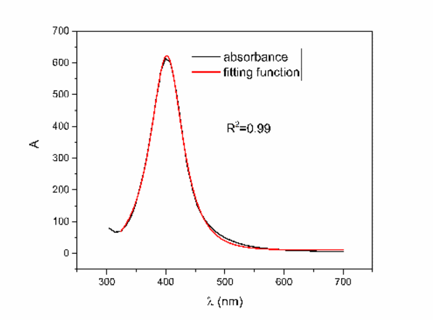	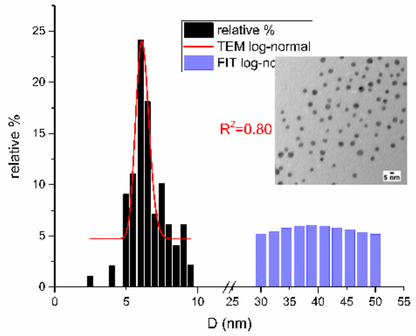
EcoX_2<br><br>D_{TEM}=(24±3)<br>D_{FIT}=(24.4±0.2)<br><br>S_{TEM}=(0.12±0.01)<br>S_{FIT}=(0.141±0.006)	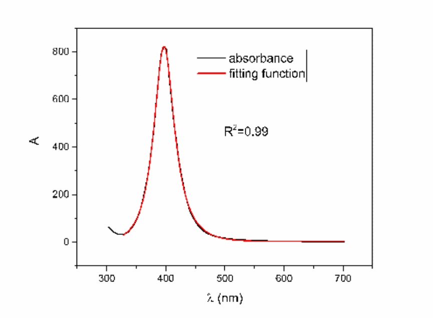	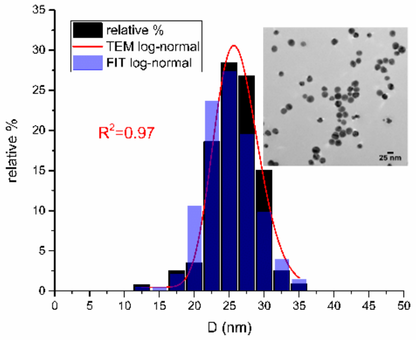

EcoX_3<br><br>$D_{TEM}=(51\pm9)$<br>$D_{FIT}=(45\pm2)$<br><br>$s_{TEM}=(0.106\pm0.008)$<br>$s_{FIT}=(0.39\pm0.06)$	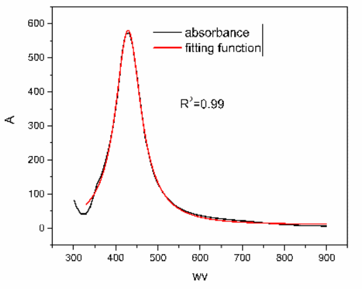	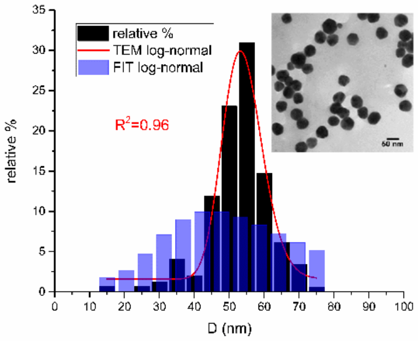
EcoX_4<br><br>$D_{TEM}=(77.5\pm7.0)$<br>$D_{FIT}=(78\pm2)$<br><br>$s_{TEM}=(0.077\pm0.005)$<br>$s_{FIT}=(0.27\pm0.02)$	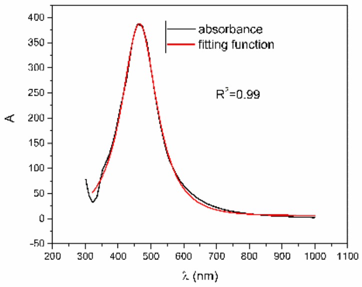	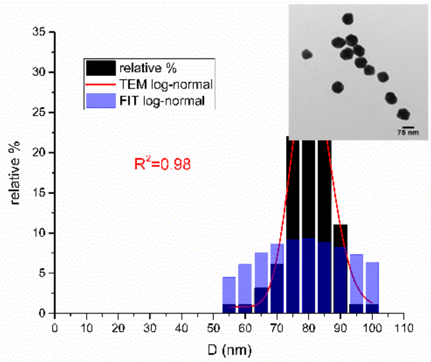
EcoX_5<br><br>$D_{TEM}=(106\pm14)$<br>$D_{FIT}=(76\pm3)$<br><br>$s_{TEM}=(0.12\pm0.02)$<br>$s_{FIT}=(0.51\pm0.01)$	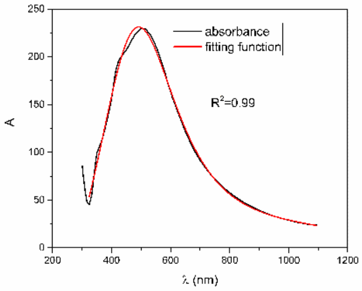	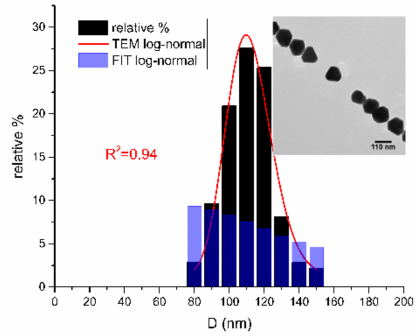
AgIn_1<br><br>$D_{TEM}=(26.3\pm0.4)$<br>$D_{FIT}=(20.7\pm0.1)$<br><br>$s_{TEM}=(0.30\pm0.01)$<br>$s_{FIT}=(0.318\pm0.002)$	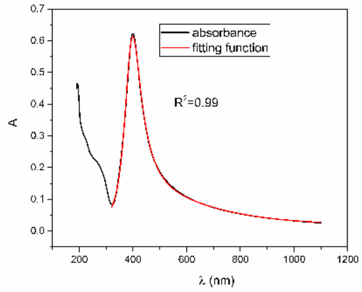	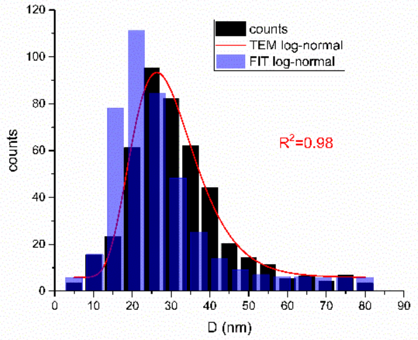

AgIn_2<br><br>$R_{TEM}=(15\pm5)$<br>$D_{FIT}=(30\pm5)$<br><br>$s_{TEM}=()$<br>$s_{FIT}=(0.43\pm0.03)$	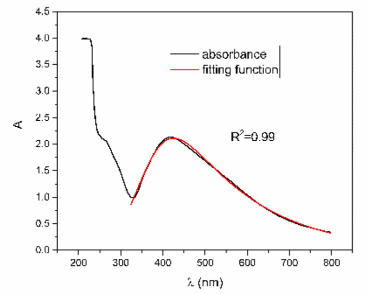	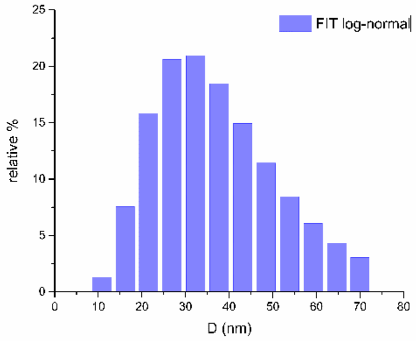
AgIn_3<br><br>$R_{TEM}=(34\pm5)$<br>$D_{FIT}=(80\pm6)$<br><br>$s_{TEM}=()$<br>$s_{FIT}=(0.52\pm0.06)$	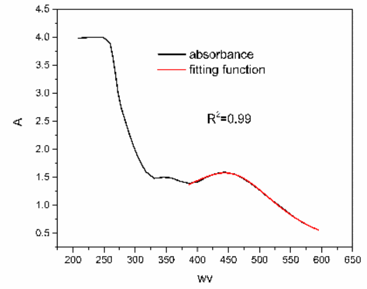	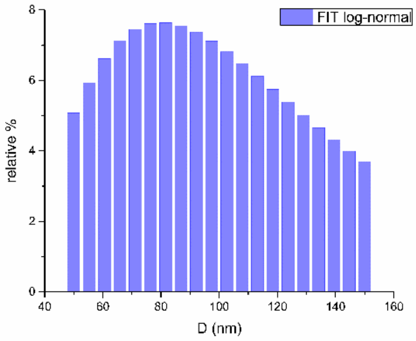

Table S3. Labels, types, shapes, reported and fitted concentrations for colloidal solutions of nanoparticles with corresponding error.

Sample no.	Label	Type	Shape	Reported Concentration ($\cdot10^{10}$ cm^{-3})	Fitted Concentration without r(D, λ) ($\cdot10^{10}$ cm^{-3})	Fitted Concentration with r(D, λ) ($\cdot10^{10}$ cm^{-3})
1	Ag_1	PLAL, 500 p	spherical	2.0	(2.3±0.4)	(1.9±0.3)
2	Ag_2	PLAL, 1000 p	spherical	3.4	(3.4±0.3)	(2.9±0.3)
3	Ag_3	PLAL, 2000 p	spherical	4.7	(6.2±0.3)	(5.2±0.3)
4	Ag_4	PLAL, 3000 p	spherical	6.7	(8.6±0.4)	(7.4±0.4)
5	Ag_5	PLAL, 5000 p	spherical	10.4	(11.9±0.7)	(10.4±0.7)
6	NanoX_1	NanoXact	spherical	370	(29.2±0.7)	(28.0±0.7)
7	NanoX_2	NanoXact	spherical	52	(30.4±0.8)	(29.8±0.8)
8	NanoX_3	NanoXact	spherical	16	(14.0±0.3)	(13.7±0.3)
9	NanoX_4	NanoXact	spherical	5.7	(4.68±0.09)	(4.58±0.09)
10	NanoX_5	NanoXact	spherical	3	(3.16±0.06)	(3.06±0.06)
11	NanoX_6	NanoXact	spherical	1.9	(1.96±0.04)	(1.87±0.04)
12	NanoX_7	NanoXact	spherical	0.95	(1.01±0.03)	(0.94±0.03)

13	NanoX_8	NanoXact	spheri-cal	0.76	(0.66±0.02)	(0.62±0.02)
14	NanoX_9	NanoXact	spheri-cal	0.43	(0.30±0.04)	(0.29±0.04)
15	NanoX_10	NanoXact	spheri-cal	0.055	(6±1)	(5±1)
16	BioP_1	BioPure	spheri-cal	120000	(301±2)	(283±2)
17	BioP_2	BioPure	spheri-cal	20000	(1810±20)	(1750±20)
18	BioP_3	BioPure	spheri-cal	2400	(1460±40)	(1440±40)
19	BioP_4	BioPure	spheri-cal	770	(790±20)	(780±20)
20	BioP_5	BioPure	spheri-cal	320	(264±6)	(259±6)
21	BioP_6	BioPure	spheri-cal	180	(134±3)	(129±3)
22	BioP_7	BioPure	spheri-cal	92	(130±2)	(123±2)
23	BioP_8	BioPure	spheri-cal	54	(61±1)	(57±1)
24	BioP_9	BioPure	spheri-cal	40	(34±1)	(32±1)
25	BioP_10	BioPure	spheri-cal	21	(8.0±0.5)	(7.6±0.5)
26	EcoX_1	Econix	spheri-cal	370000	(1640±60)	(1570±60)
27	EcoX_2	Econix	spheri-cal	6500	(6800±200)	(6600±200)
28	EcoX_3	Econix	spheri-cal	760	(1300±200)	(1100±100)
29	EcoX_4	Econix	spheri-cal	200	(220±20)	(200±20)
30	EcoX_5	Econix	spheri-cal	83	(330±30)	(220±30)
31	AgIn_1	PLAL	spheri-cal	1	(23.9±0.3)	(20.5±0.3)
32	AgIn_2	reduction of $AgNO_3$	spheri-cal	-	-	-
33	AgIn_3	reduction of $AgNO_3$	spheri-cal	-	-	-

S4. Procedure for Size Distribution Reconstruction

Size distribution is reconstructed based on 2 fitted parameters: mode diameter D_m and standard deviation of size distribution s since log-normal distribution function is defined by only those 2 parameters. As it can be seen from the main model equation (18) in manuscript, log-normal size distribution is incorporated in fitting function via concentration of nanoparticles. According to equations (3c) and (3d), log-normal distribution function is fully defined by parameters D_m (peak of distribution) and s (width of distribution). Parameter P which appears in both (3c) and (3d) represents the integral under log-normal distribution function and it is irrelevant because it cancels out in volume concentration definition (3b) which is why it's not present in fitting function. However, reconstruction of the size distribution according to (3d) doesn't depend on parameter P since only relative percentage of nanoparticles vs diameter is of interest. It means that following formula for relative percentage can be used:

$$r_i\% = \frac{f(D_i)}{\sum_{i=1}^{n} f(D_i)} \cdot 100\% = \frac{e^{-\frac{\left(\ln\left(\frac{D_i}{D_m}\right)\right)^2}{2s^2}}}{\sum_{i=1}^{n} e^{-\frac{\left(\ln\left(\frac{D_i}{D_m}\right)\right)^2}{2s^2}}} \cdot 100\% \tag{S1}$$

where n is number of intervals representing column bars in size distribution histogram and D_i is given as $D_i = D_{min} + i \cdot \Delta D$ in range $[D_{min} .. D_{max}]$. Diameter interval is given by $\Delta D = \frac{D_{max} - D_{min}}{n}$. Also, following identity of log-normal distribution function can be shown:

$$\int_0^{\infty} e^{-\frac{\left(\ln\left(\frac{D}{D_m}\right)\right)^2}{2s^2}} dD = \sqrt{2\pi} s D_m e^{\frac{s^2}{2}} \tag{S2}$$

Using property of log-normal function for condition $f(D_{min}) = f(D_{max})$, one obtains

$$D_{min} \cdot D_{max} = {D_m}^2 \tag{S3}$$

Here, D_{min} and D_{max} aren't minimum and maximum diameters of nanoparticles in colloidal solution since those cannot be deduced from fitting procedure. Values D_{min} and D_{max} represent boundary diameters for which size distribution is reconstructed because log-normal function is defined within the range $[0..+\infty]$. Using definition of ΔD and (S3), one can find expressions for $D_{min,max}$ which equal:

$$D_{max} = \frac{1}{2}\left(n\Delta D + \sqrt{n^2 \Delta D^2 + 4D_m^2}\right) \tag{S4}$$

$$D_{min} = \frac{2D_m^2}{n\Delta D + \sqrt{n^2 \Delta D^2 + 4D_m^2}} \tag{S5}$$

In summary, for defined ΔD and n one can find D_{min} and D_{max} and use above procedure to reconstruct size distribution of colloidal nanoparticles using (S1).
